# Character level attention siamese networks for Chinese handwriting comparison

**DOI:** 10.1371/journal.pone.0353503

**Published:** 2026-09-17

**Authors:** Li Zhu, Long Tang, Huaqing Mao

**Affiliations:** 1 School of Artificial Intelligence, Guangzhou Maritime University, Guangzhou, Guangdong, China; 2 School of Information Engineering, Hubei University of Economics, Wuhan, Hubei, China; 3 Hubei Key Laboratory of Digital Finance Innovation, Hubei University of Economics, Wuhan, Hubei, China; 4 Hubei Internet Finance Information Engineering Technology Research Center, Hubei University of Economics, Wuhan, Hubei, China; 5 College of Mathematics and Computer Science, GuangDong Ocean University, Zhanjiang, Guangdong, China; University of Kerbala, IRAQ

## Abstract

Handwriting recognition comparison is a critical yet challenging task in financial, commercial, and exam cheating prevention applications. However, the drawbacks of existing methods are that they compare the whole sentence or the entire signature with multiple characters susceptible to data noise. Their accuracy fluctuates widely. Unlike signature verification which treats the entire signature as a single biometric pattern, or general handwriting comparison which analyzes text at the word or paragraph level, our approach operates at the character level, enabling finer-grained analysis with reduced sensitivity to spacing and alignment variations. In this paper, we propose a character-level attention siamese network for handwriting comparison. The proposed model is a 2-channel CNNs model with multiple spatial-attention convolutional layers. Each channel receives an image of a single Chinese handwriting character. The model outputs the similarity between the two characters. Extensive experiments show that our model’s attention mechanisms and the additional information of characters can effectively reinforce valid information about signature comparisons. Specifically, the proposed CLASN+Fonts model achieves a False Acceptance Rate (FAR) of 11.93%, False Rejection Rate (FRR) of 9.36%, and Accuracy (ACC) of 90.78%, representing a 44.06% reduction in FAR, 42.6% reduction in FRR, and 16.12% improvement in ACC over the baseline SVM method. Since no suitable Chinese character signature dataset is currently available, we collected a large-scale Chinese signature dataset with approximately 48,000 handwritten character images of 800 users and 3,000 character images of 50 fonts. Further analysis of experimental results demonstrates that the proposed methods extract the comparison feature of Chinese character handwriting and select the most informative region automatically during the training.

## 1. Introduction

Handwriting comparison is a critical task in forensic analysis, financial verification, and examination security. The core challenge is to reliably determine whether two handwriting samples originate from the same individual. While existing methods have achieved notable success in signature verification for Latin scripts, Chinese handwriting comparison presents unique challenges due to the complex stroke structures of Chinese characters and the lack of specialized datasets and methods designed for character-level analysis.

Despite significant progress in handwriting verification, several critical gaps remain: Dataset Gap: Existing public datasets (CEDAR, MCYT, BHSig-260, GPDS) focus primarily on Latin script signatures at the whole-signature level. There is a lack of large-scale Chinese character-level datasets designed specifically for handwriting comparison.

Methodological gap: Current deep learning approaches typically process entire signatures or multiple characters together, making them susceptible to noise from character spacing, rotation, and background variations. Character-level processing has not been adequately explored.

Feature disentanglement gap: Existing methods struggle to separate handwriting style features from character structure features, particularly for Chinese characters with complex stroke patterns.

Attention application gap: While attention mechanisms have shown success in NLP and general computer vision, their application to character-level handwriting comparison remains underexplored.

Can a character-level attention-based Siamese network effectively capture discriminative stroke features for Chinese handwriting comparison, and will incorporating character identity information through one-hot encoding improve the model’s ability to focus on handwriting style rather than character structure? We hypothesize that: (1) Processing handwriting at the character level (rather than whole signature) will reduce sensitivity to noise factors such as character spacing and rotation. (2) Incorporating spatial attention mechanisms will enable the model to focus on the most discriminative stroke regions. (3) Providing character identity through one-hot encoding will help disentangle handwriting style features from character structure features, leading to more accurate comparison.

In this work, we analyzed the factors affecting handwriting recognition results and inspired by the tremendous success of attention mechanism in natural language processing [1–3] and convolutional neural networks [4–8]. We proposed a siamese network at the character level that trains on a large-scale Chinese character signature dataset to solve the Chinese handwriting recognition comparison task. For simplicity, we denote the proposed character level attention siamese network and Chinese character signature dataset as CLASN and CCSD. The CLASN consists of a pair of feature extractors with a spatial attention mechanism and a similarity calculation module. The feature extractor uses CNN with attention to extracting features of a single character. Here is the pair of CNNs using the siamese networks with the identical architecture. Therefore, the proposed model can process two input characters simultaneously. Furthermore, the spatial attention mechanism considers the contributions of a different region of the signature picture when the model inputs different characters. Moreover, we build a new large-scale and challenging Chinese character signature dataset and randomly generated the training dataset with positive sample pairs and negative sample pairs to significantly increase the amount of data. We also demonstrate our model achieve a reduction of 44.06% FAR, 42.6% FRR, and an improvement of 16.12% ACC over the baseline SVM.

The contributions of this paper can be summarized as three folds: We apply the character level information to Chinese handwriting recognition comparison, which reduces the impact of noise on results. We propose attention siamese networks with dual CNN architectures, which capture the feature of Chinese character handwriting and select the most informative region automatically during the training. We build a new large-scale CCSD and collect approximately 48,000 handwritten Chinese character images of over 800 users and 3,000 character images of 50 fonts.

The whole paper is organized as follows. Section Related Work introduces the related work. In Section Attention Siamese Networks, we describe our proposed model. We present the dataset and the experiments and make analyses and discussions in Section Experiments. Finally, in Section Conclusion and Future Work we conclude this paper and explore the future work.

## 2. Related work

### 2.1 Traditional machine learning methods

The most typical traditional machine learning method for handwriting comparison is the KNN algorithm. The algorithm first transforms the image that needs to be compared into a matrix, finds the K neighbors in training set closest to the matrix, and determines the type of the new data based on their main classification. The main classification can be based on different criteria, such as “most numerous” “Euclidean distance” or “weighted distance.”

**Fig 1:**
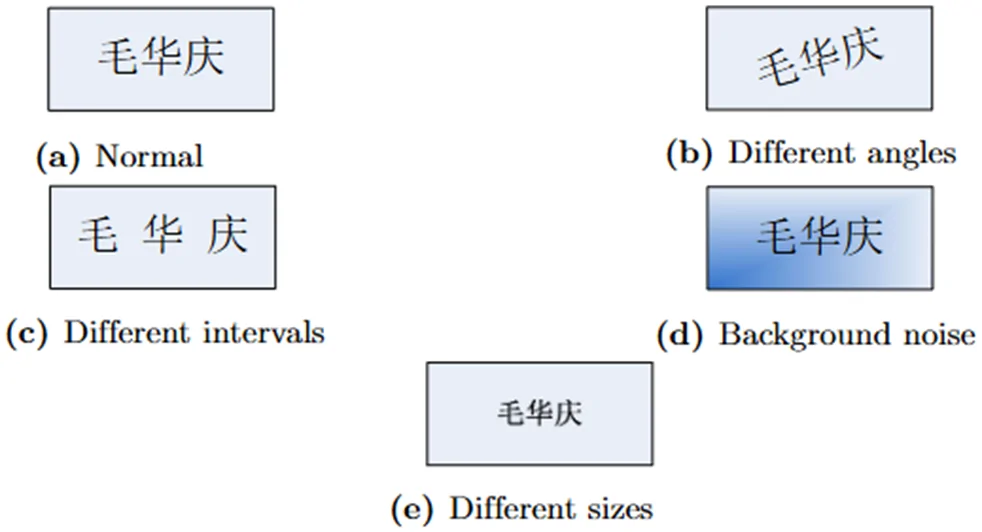
Typical noise effects of signature images. For comparison, (a) is a standard signature image, the signature in (b) has rotation, the characters in (c) have a larger interval, the background in (d) has noise due to scanning or photography, the size of the characters in (e) is not the same as the normal size, and of course, some signatures that in many of these situations at the same time. The Chinese characters shown in the signature images read “Mao Huaqing,” which is the name of one of the authors.

In the KNN algorithm [9] for handwriting style identification, N×N signature images are usually used as input. The position, angle, spacing, and signature size can cause significant errors(shown in Fig 1). Since the proportion of signature in the image is relatively small (the information used for discrimination is sparse when the image is converted to a matrix), the gray-scale value, font color, and stroke thickness from the original scanned image have an enormous impact on the discrimination results. Therefore, the KNN algorithm requires complex pre-processing of the image: resizing, filtering, and color grading to get a good result. In particular, the KNN algorithm is less efficient when the number of images compared in the training data is large. There are other traditional machine learning algorithms such as SVM [10], LBP [11], and GMM [12] besides KNN.

Critical analysis of traditional methods:

- Strengths: KNN and SVM are computationally efficient, interpretable, and work well with limited data.
- Limitations: They require extensive preprocessing (resizing, filtering, color grading); are particularly sensitive to character rotation, variable spacing, and background noise; have limited feature learning capability; and show poor scalability to large datasets.
- Why Insufficient: These factors are common in real-world Chinese handwriting, making traditional methods unreliable for character-level comparison.

### 2.2 Deep learning methods

Relative to traditional machine learning algorithms, deep neural networks can learn features autonomously and have achieved excellent results in face recognition, object detection, and identification applications. Some neural network models have also been used for handwriting recognition comparison [13,14]. A commonly used unsupervised model for image feature extraction is auto-encoder, which automatically acquires an image’s feature representation to minimize the reconstruction error between encoder and decoder. The deep neural network model also has two deficiencies in the handwriting recognition and comparison task. The first is that each person writes a different character. When the model automatically acquires the feature representation of the picture, it acquires the handwriting information. It learns additional information about the text itself, which is not helpful for handwriting comparison but makes it more difficult for the model to distinguish handwriting. Also, a general deep neural network can only handle a single input of an image, which is less efficient in scenarios where two or more images are compared.

**Fig 2:**
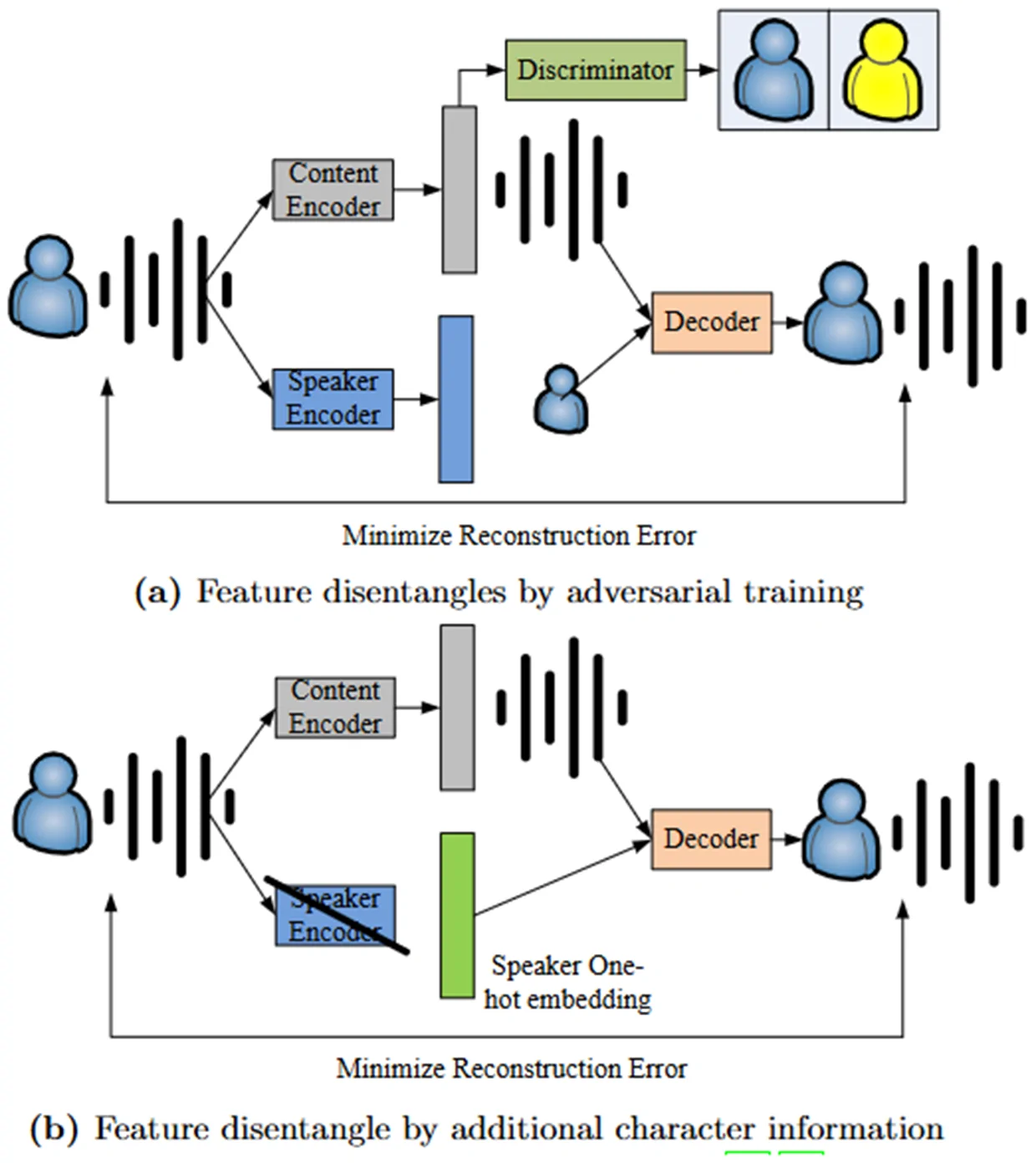
Two feature disentangle methods [15,16] applied in voice conversion, the adversarial training method is more generalized, but the complex architecture is difficult to train; another method is easy to implement, but it can not process the voice never present in the training set, so it has less generalization ability.

For the first issue, there are two solutions for feature disentangle, and one is to use the concept of adversarial training [15], add additional text classifiers as the discriminator, and add a training goal for the encoder, which is to make the discriminator can not distinguish the character, the model structure is shown in Fig 2a.

The solution is relatively complex, which requires iterative training of the discriminator and encoder and its convergence is difficult. Another solution is adding character information to the model [16]. The information can represent by one-hot encoding. Since training data already provide additional character information, encoder and decoder are trained not to involve character context information (Shown in Fig 2b. However, this solution cannot handle new characters, and for those characters that are not in the training data, the UNK(Unknow character) is not suitable for this situation. As the characters to be compared in the signature comparison task are known in advance, it is most convenient to use this solution to filter the character context information and focus on the handwriting feature extraction.

The second problem can be solved using a siamese network that can process two inputs simultaneously. Siamese has two neural networks (CNN/LSTM/RNN) with the same structure and shared weights, which can acquire the feature vectors of both inputs simultaneously and then construct a contrastive loss function employing a distance metric to determine the similarity and train the network. Siamese network has a wide range of applications, initially mainly used for signature authentication identification. However, later it has many applications in both CV and NLP fields, such as face verification [17], handwriting recognition [18], visual tracking algorithm [19], semantic similarity analysis of the sentence [20], matching of question and answer [21]. In the *Kaggle* kernel competition called *Quora Insincere Questions Classification*, the siamese network determines whether two questions are the same question. Since its origin as one of the earliest siamese applications, handwriting authentication has been plentiful research. Many related word-based signature datasets have been published, e.g., CEDAR [22,23], MCYT-75/100 [24], BHSig-260 [25,26], and GPDS [27,28]. Those signatures in English are split by word, but a signature usually contains more than one Chinese character [29]. There are also some public Chinese signature datasets, such as SigComp11 [30], CSD [31], GTR [32] etc. The signatures in these datasets are based on multiple characters. Therefore, training from the character level requires re-collecting data to mitigate the adverse effects of noise.

Critical analysis of deep learning methods:

- Strengths: CNNs can automatically learn features, show better robustness to variations, and are scalable.
- Limitations: They are data-hungry, have black-box nature, and struggle with feature disentanglement.
- Why Insufficient: Standard CNNs typically process entire signatures or multiple characters together, learning both handwriting style AND character structure simultaneously, which leads to confusion when comparing characters with similar structures but different handwriting styles.

### 2.3 Attention mechanism

The attention mechanism was inspired by brain signal processing mechanisms specific to the human visual system(HVS). The human vision quickly scans the global image to obtain the target area to be focused, known as the attention zone, and then devotes more additional resources to this area to obtain more details about the target and block out other unimportant information. Initially used for machine translation [3], attention mechanism has been widely used in recent years in various areas of NLP, such as speech recognition [33], recommendation system [34], text comprehension [35], document classification [36].

The attention mechanism also has many applications in computer vision, where its primary purpose is to allow deep neural networks to learn the regions of each image that need attention. There are two different types of attention mechanisms in computer vision: soft attention and hard attention. In particular, soft attention mechanisms are divided into: regional attention networks, channel attention networks, and hybrid domain attention networks. Soft attention is deterministic attention, which can be generated directly by the neural network after training. The critical point is that soft attention is differentiable, so the gradient can be calculated by the neural network and the forward propagation and backward feedback can be used to learn the attention weights [37]. The idea of hard attention [38], unlike soft attention, is that every point in an image has the potential to be attended to, and hard attention is a stochastic prediction process with more emphasis on dynamic changes. The crucial thing is that hard attention is non-differentiable and the training process is often trained by reinforcement learning. Also, there are many attention mechanisms related to research, such as residual attention network [39], interaction-aware spatiotemporal pyramid attention networks [40], recursive attention mechanisms [41], and cross-attention mechanism [42,43].

### 2.4 Comparison with recent studies

Recent advances in handwriting verification have demonstrated the effectiveness of deep learning approaches. Chao-Qun et al. proposed an offline handwriting verification method based on Siamese networks with multi-channel fusion, achieving state-of-the-art performance on Chinese handwriting datasets [44]. Their dual-channel architecture shares similarities with our CLASN, but our approach incorporates spatial attention mechanisms and character one-hot encoding for better feature disentanglement.

Liu et al. developed a multi-scale SE attention model for handwritten Chinese character recognition, achieving 98.6% accuracy on writing grade examination datasets [45]. While their recognition task differs from our comparison task, their attention mechanism design validates the effectiveness of attention-based approaches for Chinese character analysis.

Kulkarni & Wagh proposed a Transfer Learning-enhanced Siamese Neural Network for signature verification, achieving 90.10% accuracy with EER of 10.08% [46]. Our CLASNF model achieves comparable performance (90.78% ACC, estimated EER 10.6%) on the more challenging character-level comparison task.

Afzali et al. developed a Customized Siamese CNN for writer verification using combined loss functions, demonstrating the effectiveness of Siamese architectures across different languages (English and Arabic) [47].

Huang et al. proposed MS-SigNet with co-tuplet loss for offline signature verification and introduced HanSig, a large-scale Chinese signature dataset [48]. While their work focuses on signature-level verification, our CCSD dataset is specifically designed for character-level Chinese handwriting comparison.

## 3. Attention siamese networks

### 3.1 Overview

In this section, we will introduce our proposed methods for handwriting comparison tasks in detail. First, we present the overview of the model in Section Overview. Second, we give the detail of the proposed siamese networks in Section Siamese networks architecture. Finally, Section Attention explains the attention mechanism in the model.

**Fig 3:**
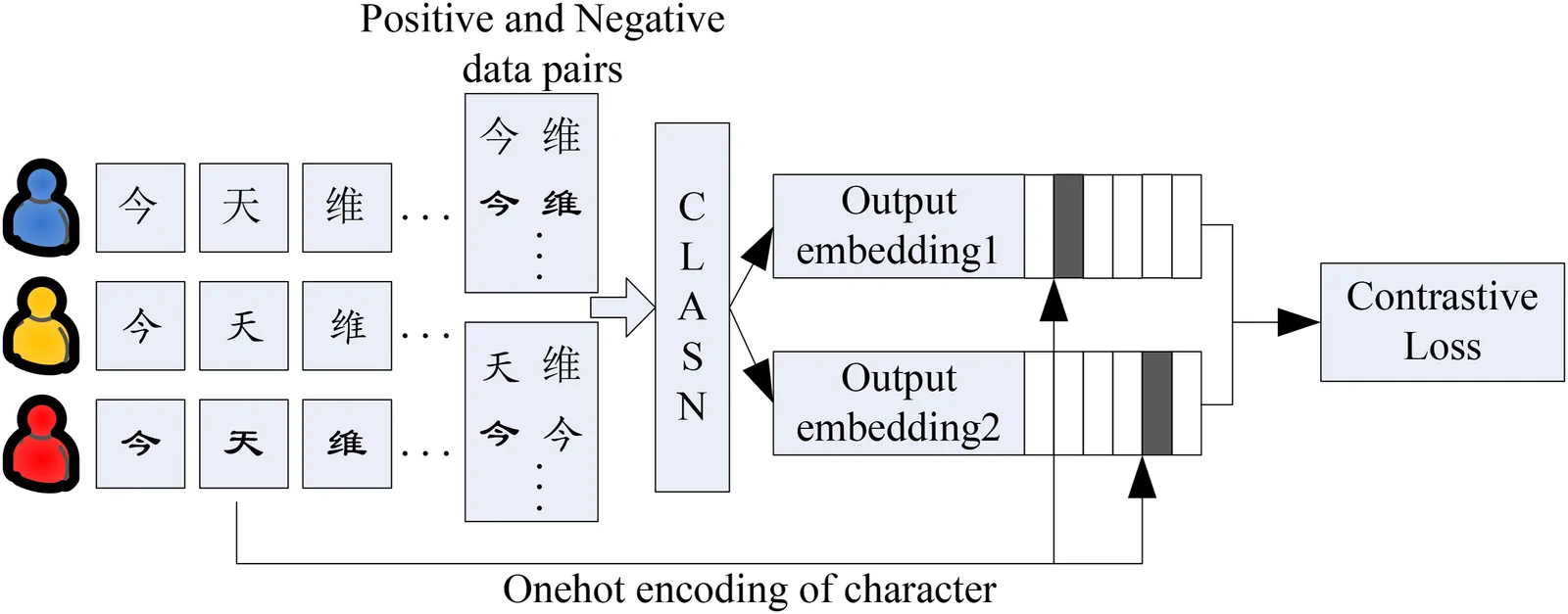
The overview of our proposed method. In the examinations, candidates need to write down the same content character by character in the squares of the test paper, and the handwriting comparison is used to identify the cases of surrogate exam-takers. The figure shows handwriting samples from three different candidates; each sample contains the same three Chinese characters arranged from left to right. The first character (leftmost) is jin (meaning today or now), the second is tian (meaning day or sky), and the third (rightmost) is wei (meaning dimension or to maintain).

We will start by defining some notations and describe the handwriting comparison task. Our model accepts two Chinese character images as input. The task is to determine whether those two character images come from the same person. Unlike traditional handwriting verification tasks, our task is base on character instead of multiple words or phrases. The handwriting comparison task can be model as finding the similarity of two images of the character. The Siamese architecture is chosen because handwriting comparison is fundamentally a similarity learning problem. By using two identical networks with shared weights, we ensure that: (1) The same feature extraction process is applied to both inputs, (2) The learned embeddings are directly comparable, (3) The model can generalize to unseen user pairs. An overview of our proposed method is shown in Fig 3. Firstly, we generate the list of data pairs. The number of positive data pairs is approximately equal to the number of negative data pairs, which can avoid the unbalanced data problem. Then we divide the training, validation, and test datasets in the list. Secondly, after we feed the paired sample data into the model, we get two images’ output embedding. Thirdly, we concatenate the one-hot encoding of character to the embedding and calculate the Euclidean distance between data pairs’ results. Finally, the model determines whether the same user writes two characters according to the margin value *r*, which is the hyper-parameter relate to the error rate.

### 3.2 Siamese networks architecture

The Siamese architecture is chosen because handwriting comparison is fundamentally a similarity learning problem. By using two identical networks with shared weights, we ensure that: (1) The same feature extraction process is applied to both inputs, (2) The learned embeddings are directly comparable, (3) The model can generalize to unseen user pairs.

**Fig 4:**
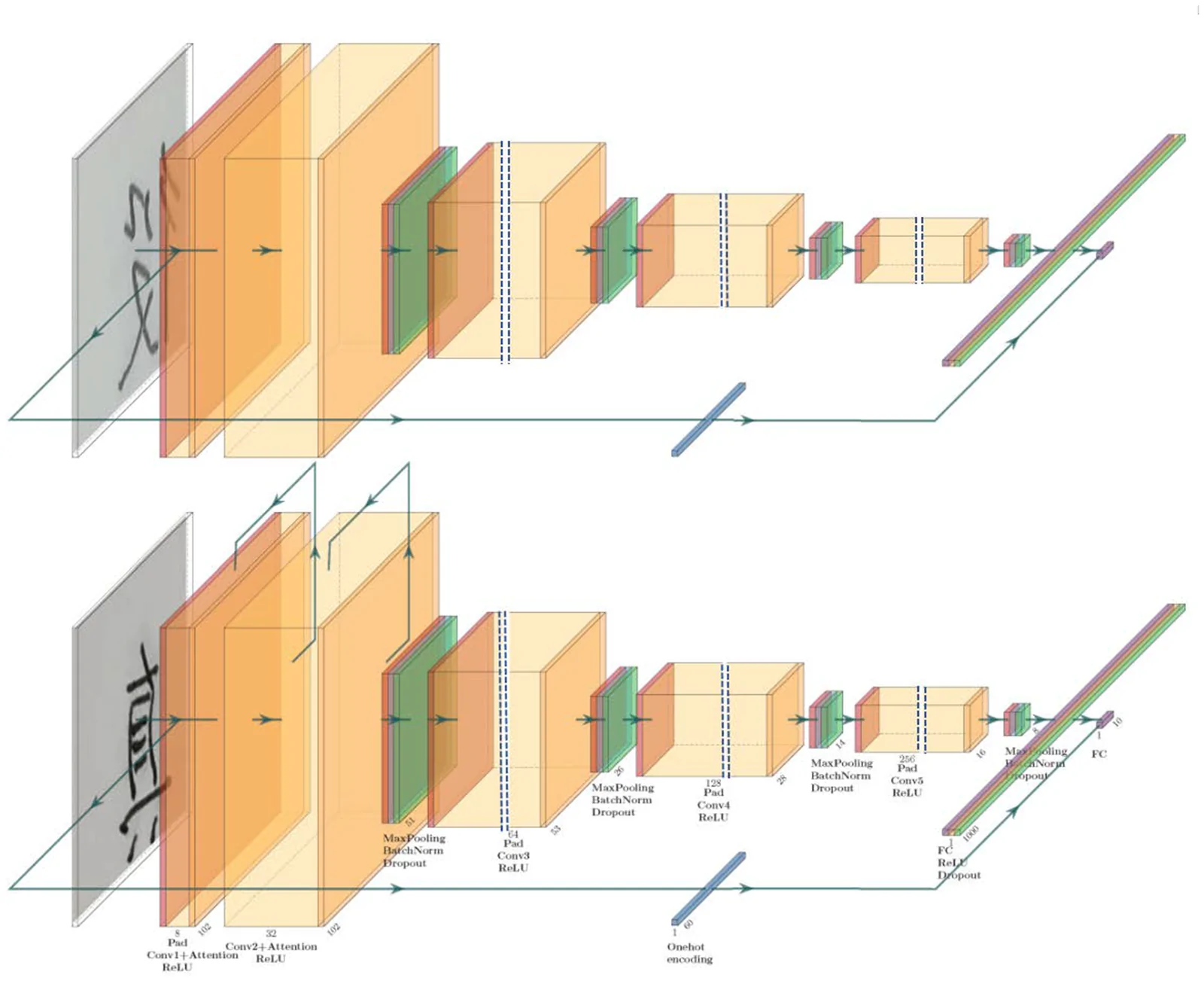
An illustration of the architecture of CLASN explicitly showing the forward propagation of two Chinese character images. The one-hot encoding of the character is concatenated to the output embedding before we calculate the contrastive loss. Those two channels share weights, and the input of each channel is 100×100 dimensional, and the parameter number of 5 convolutional and 2 fully connected layers is given by 80/2,336/18,496/73,856/295,168/16,385,000/10,010. We will show the specific details of Conv1 and Conv2’s attention operation in Section Attention.

The main idea of the siamese network used in this paper is to map the input images to the target space by a series of convolutional layers with spatial attention mechanism and compare the similarity in the target space using simple distances metrics (Euclidean distances, cosine distance, or Manhattan distance) [49]. In the training phase, the goal is to minimize the value of the loss function for a pair of character images from the same user and maximize the value of the loss function for many samples from different users. The siamese network architecture is shown in Fig 4, which is a 2-channel convolutional neural network. After each convolutional layer, we use the batch normalization layer to prevent vanishing gradient and speed up the training process; and we use the dropout layer to prevent overfitting. Although two CNNs are used to extract character features in the architecture, they are equivalent to one network because they share weights.

As depicted in Fig 4, the network contains seven layers with weights; the first five are convolutional, and the other two are fully connected. The spatial attention mechanism of the convolutional layer will be detailed in Section Attention. The output of the last fully connected layer is concatenated to the one-hot encoding of the input character. We apply the ReLU non-linearity to the output of every convolutional and fully connected layer, except the last layer. After every convolutional layer besides the first one, We employ the max pooling, batch normalization, and dropout layers throughout our network, with *pooling kernel size* = 2, *stride* = 2, *dropout rate* = 0.2. To prevent the loss of handwriting information near the edges of the image, we perform a reflection pad operation before each convolutional layer, with *padding size* = 1. All five convolutional layers have the same kernel size 3×3 with a stride of 1 *pixels*. The convolutional kernels in these convolutional layers are 8, 32, 64, 128, 256, respectively. The two fully connected layers have 16384 and 10 neurons, and the outputs of them are 1000 and 10 each. Since the character table in our dataset has 60 characters, the one-hot encoding of character is 60 dimensions which can be expressed as $x^{enc}$. Character one-hot encoding is inspired by feature disentanglement research [18]. By explicitly providing character identity, we enable the network to: (1) Filter out character structure information, (2) Focus exclusively on handwriting style features, (3) Compare characters with different structures more effectively.

Assume that there are a total of *N* users involved in the dataset, and each person has a character table containing 60 characters. We can donate the input data as:


$$\begin{array}{cc} S=\{ & (x_{1},y_{1}),(x_{2},y_{1}),\cdots ,(x_{60},y_{1}),\\ & (x_{1},y_{2}),(x_{2},y_{2}),\cdots ,(x_{60},y_{2}),\\ & \;\vdots \\ & \left(x_{1},y_{N}),(x_{2},y_{N}),\cdots ,(x_{60},y_{N})\right\} \end{array}$$ (1)

where $x_{i}(i=1,2,\cdots ,60)$ is the grayscale handwriting image with size=100×100 and $y_{n}(n=1,2,\cdots ,N)$ is corresponding writer.To simplify the notation, we donate the character image as:


$$\begin{array}{c} X=x_{i}^{n},i=1,2,\cdots ,60,n=1,2,\cdots ,N \end{array}$$ (2)

$D_{ij}$ is shot notation for $D_{ij}(⃗X_{i},{\vec{X}}_{j})$, it represents the distance between paired image *i* and *j* and it can be calculated as follows:


$$\begin{array}{cc} D_{ij} & =D_{ij}(⃗X_{i};{\vec{X}}_{j})\\ & =||[G_{\theta }(⃗X_{i});⃗X_{i}^{enc}]-[G_{\theta }(⃗X_{j}),⃗X_{j}^{enc}]|{|}_{2} \end{array}$$ (3)

where *G* represents the siamese network with parameters θ, the output embedding of network $G_{\theta }(⃗X_{i})$ is concatenated with the one-hot encoding of i-th character $⃗X_{i}^{enc}$. The distance calculation method can choose *L*_2_ norm distance.

With the distance, we can use pair-based metric learning loss to train the model. Experiments show that the *softmax* loss function in the handwriting recognition MNIST test set (handwriting digital image set) exhibit little discrimination boundary, so we use *contrastive* loss [50] function to process the paired data as follows:


$$\begin{array}{c} L(\{D_{ij}\})=\sum_{y_{n}=y_{m}}D_{ij}+\sum_{y_{n}\neq y_{m}}[r-D_{ij}{]}_{+} \end{array}$$ (4)

where *r* is margin, when the input data is a negative sample pair($y_{n}\neq y_{m}$), if the distance between two samples is greater than *r*, the model loss value will be 0, implying that the model is not updated, and $y_{n}=y_{m}$ mean the two input character is written by the same user (Positive data pair). Vice versa means that they were written by different users(Negative data pair). []_+_ is hinge function, which can be expressed as:


$$\left[x{]}_{+}=max(0,x\right)$$ (5)

### 3.3 Attention

**Fig 5:**
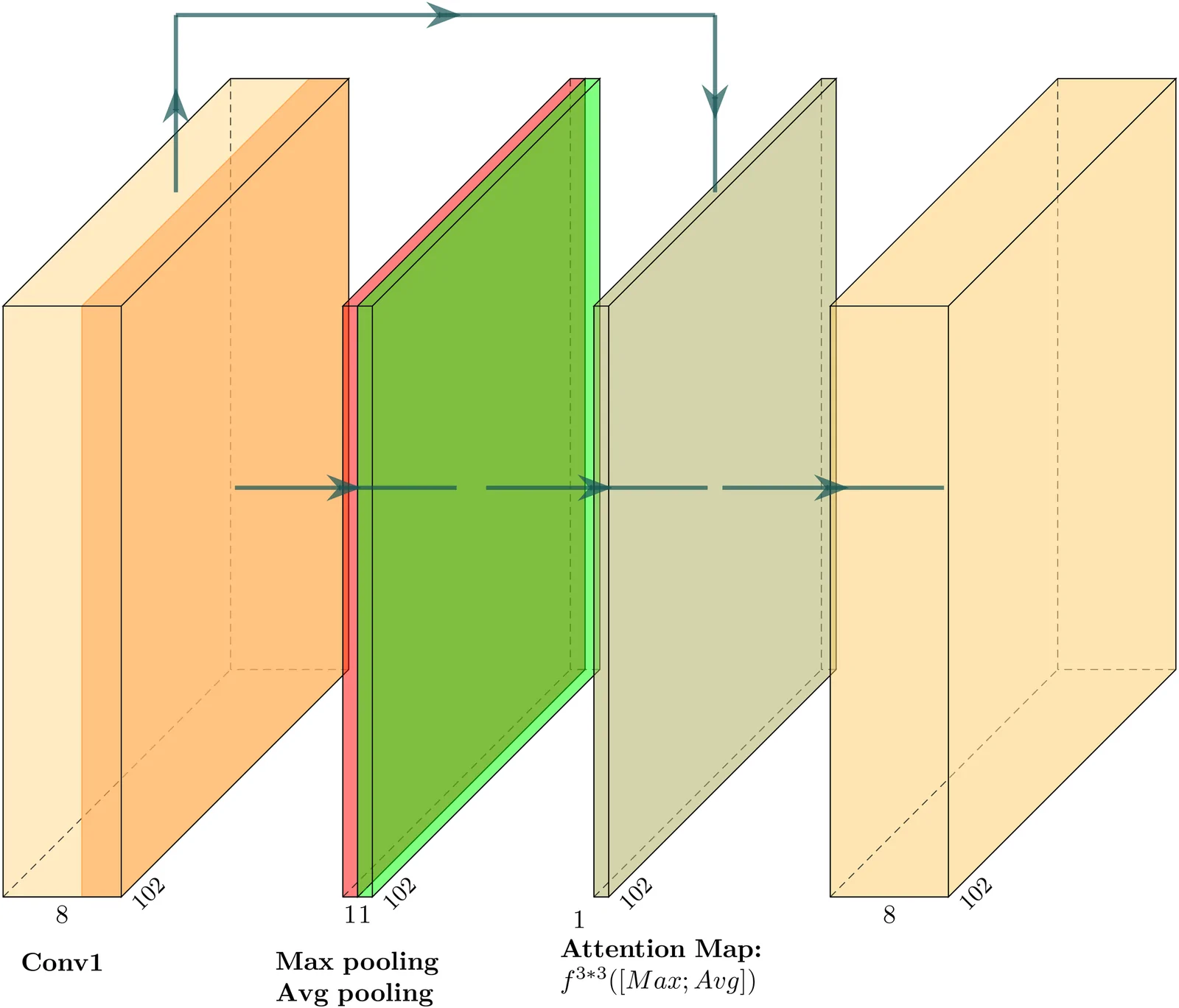
The spatial attention operation of Conv1. After the attention operation, the output turns out to have the same shape size as Conv1.

Following the previous work [51], we adopt the convolutional block attention module(CBAM) as our attention mechanism. A channel and spatial-based cascading attention mechanism are used in CBAM to enhance the feature map’s valuable features and suppress the useless features so that the model can focus on the critical regions of different input data. The channel attention module focuses on the meaningful feature map; The spatial attention module focuses on the informative part. Spatial attention is chosen over channel attention because: (1) Character images are grayscale (single channel), making channel attention less informative, (2) Different strokes within a character have varying discriminative power, (3) Attention maps provide interpretability by highlighting important regions. Since our character comparison task processes a grayscale image of characters, which contains only one channel, we ignore the channel attention module and directly perform spatial attention operations on the results of the first two convolutional layers in siamese networks. The specific steps are shown in Fig 5.

Firstly, we apply the global average pooling and global max-pooling in the channels’ dimension, and we concatenate two h×w×1 pooling feature maps ($\left[F_{avg}^{s};F_{max}^{s}\right]$) from the h×w×c shape input *F*. To keep the output shape the same as the input shape, the kernel size we can use is 3×3 or 7×7. Here we choose the former option, which can reduce the number of parameters and computational complexity. Then we use the 3×3 convolution kernel to generate a h×w×1 spatial attention map $M_{s}(F)$. Finally, we multiply the input with the obtained new feature maps to get the spatially adjusted feature maps, which can be calculated as:


$$\begin{array}{cc} FM_{s}(F) & =F\sigma \left(f^{3\times 3}\left([AvgPool(F);MaxPool(F)]\right)\right)\\ & =F\sigma \left(f^{3\times 3}\left([F_{avg}^{s};F_{max}^{s}]\right)\right) \end{array}$$ (6)

## 4. Experiments

In this section, we evaluate our proposed model on CCSD. Firstly, we introduce the dataset and the method of data augmentation. Then we describe the evaluation metrics and baselines briefly. Finally, we analyze and discuss the experimental results in detail. Our experiments show that the CLANS model can extract the relevant features of stroke from the character image instead of the character’s meaning or structure. The extracted character embeddings are robust and have a long gap when the model discriminates the negative paired samples. The positive paired samples should be pretty close, even if they come from different characters.

### 4.1 Data augmentation and dataset

**Fig 6:**
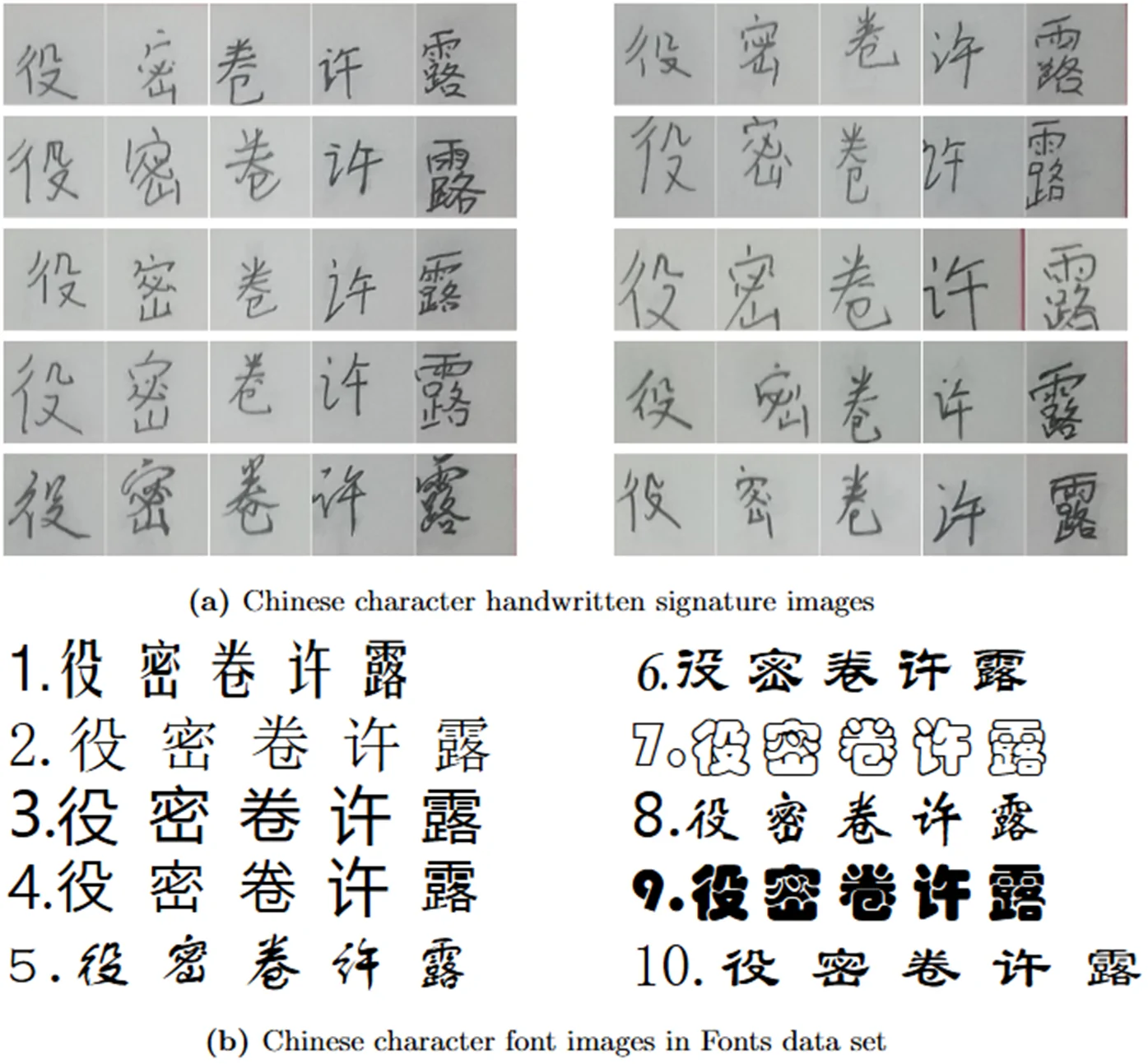
The Chinese character image in CCSD. (a) is the Chinese character signature image written by different users; (b) is examples of characters image from different fonts, each font has 60 characters. The five Chinese characters displayed in all samples are arranged from left to right as follows: the first is yi (meaning to serve or labor), the second is mi (meaning secret or dense), the third is juan (meaning volume or scroll), the fourth is xu (meaning to allow or permit), and the fifth is lu (meaning dew). Those 10 examples of Chinese fonts by serial number are FZYaoti, SimSun, Microsoft YaHei, Malgun Gothic, FZShuTi, STLiti, STCaiyun, STXinwei, STHupo, and LiSu.

We had 800 users write all the same 60 characters onto the paper within squares, then scanned and cut by squares, and some of the results are shown in Fig 6a. After getting the raw data, we still need to perform data augmentation. Data augmentation is a typical operation in the CV field, and it is the simplest and most direct way to improve model performance. Standard data augmentation methods include geometric transformations such as cropping, flipping, rotating, scaling, warping, pixel scrambling, adding noise, illumination adjustment, contrast adjustment, sample summation or interpolation, and segmentation patches. Generally speaking, the purpose of data augmentation is to extend the sample distribution space of the original dataset, not to spoil it; for example, flipping the handwriting-recognized image and then training it will reduce the effect. In this paper, we use two approaches to extend the original dataset.

The first method is shown in Fig 6b, which uses the different fonts to generate 60 characters according to the character table. We regard the same font’s characters as written by the same user and the characters of different fonts as written by different users. The second method is to use positive and negative sample pairs instead of random sampling. Instead of dividing the training, validation, and testing datasets directly by character images, the datasets are divided by users, and then $N_{pos}$ image pairs are sampled as positive sample pairs ($\{x_{i}^{n},x_{j}^{m}\},n=m$) for identical users, and $N_{neg}$ image pairs are sampled as negative samples ($\{x_{i}^{n},x_{j}^{m}\},n\neq m$) for different users. Since the sample pairs can be different permutations, the number of training samples can be greatly increased. When the number of positive and negative sample pairs is approximately equal($N_{pos}\approx N_{neg}$), the problem of data imbalance can be avoided. Besides, we can use the same raw data to generate positive and negative sample pairs of different magnitudes to perform comparative tests on the model’s scalability. The paired data generation pseudocode is shown in Algorithm 1.

**Algorithm 1. Paired data generation algorithm**

**Input:**

Input1: *userList* includs all users in the dataset

Input2: *negUserNum* is the number of negative random user samples

Input3: *negCharNum* is the number of negative random character samples

**Output:**

Output1: *posDataList*

Output2: *negDataList*

1:  **for** $user_{i}\;in\;userList$ **do**

2:   *userCharList* = getCharList($user_{i}$)

3:   //Iterate over each character for each user

4:   **for** *i* = 0; *i* < len(*userCharList*); *i*++ **do**   //Positive paired data generation

5:    **for** *j*=0; *j*<len(*userCharList*); *j*++ **do**

6:     Add (*userCharList*[*i*],*userCharList*[*j*]) to *posDataList*

7:    **end for**

8:    //Negative paired data generation

9:    **for** *k*=0; *k* < *negUserNum*; *k*++ **do**

10:     $negUserList_{k}$ = randomSampleUser(*userList*)

11:     **for** *n* = 0; *n* < *negCharNum*; *n*++ **do**

12:      $negCharList_{n}$ = randomSampleChar($negUserList_{k},userCharList$)

13:      Add $\left(userCharList[i],negCharList_{n}\right)$ to *negDataList*

14:     **end for**

15:    **end for**

16:   **end for**

17:  **end for**

18: **return** *posDataList*,*negDataList*

There are approximately 800 individuals and different kinds of the character of 50 fonts in our Chinese character signature dataset, and We divide the CCSD into training, validation and test sets by user and font. The statistics of the CCSD division are shown in Table 1.

**Table 1:** Statistics of original data. Train Set, Validation Set, Test Set denote the number of users or fonts in those datasets, respectively. Total Image is the total Chinese character image number belong to the user or font. The symbol “*” indicates that the invalid or blank images are excluded.

Character source	Train Set	Validation Set	Test Set	Total Images
Users	600	100	100	48000*
Fonts	50	0	0	3000

### 4.2 Evaluation metrics and experimental setup

Since the handwriting comparison is a special case of handwriting verification, we choose the false acceptance rate (FAR) and the false rejection rate (FRR) instead of the character error rate (CER) and character accuracy rate (CAR) [52,53]. To measure the model’s quality, we follow the previous works [31,54,55] and use FAR, FRR, and ACC (Accuracy) to evaluate our method and compare it with other existing methods. FAR is the measure of the likelihood that the model will incorrectly accept an impostor as normal data. The FAR of the model typically is stated as the fraction of the number of false acceptances divided by the number of all impostor attempts. FRR is the measure of the likelihood that the model will incorrectly reject normal data. The FRR of the model typically is stated as the fraction of the number of false rejections divided by the number of identification attempts. ACC is the most common evaluation metric in face or fingerprint recognition tasks. The ACC of the model is stated as the fraction of the number of samples classified correctly divided by all samples.

To train the proposed CLASN model, we employ the Adam optimizer with contrastive loss (margin *r* = 2.0). Given the importance of distinguishing different users’ handwriting patterns, we adopt an asymmetric sampling strategy where negative samples outnumber positive samples by a 1.5:1 ratio, which enhances the model’s discriminative capability for forgery detection. Following standard practices, input images are normalized and resized before feeding into the network, and dropout regularization is applied to prevent overfitting. We train the model for 2000 epochs and select the checkpoint with the best validation accuracy as the final model for testing.

While ROC curves and EER (Equal Error Rate) are commonly used in biometric verification, we note that FAR and FRR inherently capture the same trade-off information. Specifically, FAR corresponds to False Positive Rate (FPR) and (1-FRR) corresponds to True Positive Rate (TPR) in ROC analysis. Based on our reported results in Table 2, the approximate EER values can be estimated as the midpoint between FAR and FRR: CLASN achieves approximately 11.4% EER (midpoint of 12.72% FAR and 10.17% FRR), and CLASNF achieves approximately 10.6% EER (midpoint of 11.93% FAR and 9.36% FRR). These values are competitive with recent signature verification studies [48].

**Table 2:** Comparison between the models mentioned on CCSD. FAR, FRR, ACC denote False Acceptance Rate, False Rejection Rate, and Accuracy, respectively. The symbol “+” indicates that the higher the value is the better the model performs. The symbol “-” is the opposite.

Model	FAR(-)	FRR(-)	ACC(+)
SVM	21.33	12.64	78.18
CSN	19.52	10.23	81.67
SCSN	15.34	10.17	85.91
Baseline(no attention, no encoding)	18.42	14.23	83.67
+Spatial Attention	15.18	12.45	86.19
+Character Encoding	14.67	11.82	86.76
+Both(CLASN)	12.72	9.36	88.44
+Fonts(CLASNF)	**11.93**	**7.25**	**90.78**

### 4.3 Result

The experimental results of our model against other models on CCSD are shown in Table 2. The results show that our proposed model achieves the best performance in the main evaluation metrics we mentioned in Section Evaluation Metrics and Experimental Setup. Specifically, the ablation studies demonstrate that each proposed component contributes significantly to the final performance.

Beginning with the baseline model (without attention mechanism and character encoding), it achieves 18.42% FAR, 14.23% FRR, and 83.67% ACC. When incorporating the Spatial Attention module alone, the model obtains a reduction of 3.24% FAR, 1.78% FRR, and an improvement of 2.52% ACC. Similarly, adding Character Encoding individually yields a reduction of 3.75% FAR, 2.41% FRR, and an improvement of 3.09% ACC. Notably, when both components are integrated (CLASN), the model achieves a reduction of 5.70% FAR, 4.87% FRR, and an improvement of 4.77% ACC over the baseline, indicating that the spatial attention and character encoding modules have complementary effects.

The proposed CLASN model enhance by the Fonts dataset achieves a reduction of 44.06% FAR, 42.6% FRR, and an improvement of 16.12% ACC over the baseline SVM. Additionally, compared with traditional deep learning models such as CSN and SCSN, our CLASN model also shows substantial improvements, and the complete CLASNF model further widens this gap. Furthermore, our model outperforms the traditional deep learning model by a large margin. For example, our proposed CLASN model enhance by the Fonts dataset gets a reduction of 38.88% FAR, 29.13% FRR, and an improvement of 11.15% ACC over the CNN-based siamese model. It is worth noting that even without the Fonts dataset, our proposed CLASN model (12.72% FAR, 9.36% FRR, 88.44% ACC) still outperforms all baseline methods including SVM, CSN, and SCSN, validating the effectiveness of the proposed attention and encoding mechanisms.

Moreover, the CLASN model is remarkably improved by additional Fonts dataset. The CLASN model with Fonts data set receives a reduction of 6.21% FAR, 22.54% FRR, and an improvement of 2.65% ACC on the CCSD test set contrasted with the CLASN without Fonts dataset. This confirms that the Fonts dataset provides valuable supplementary information for cross-domain text recognition, particularly in reducing false rejections.

### 4.4 Analysis and discussion

**Fig 7:**
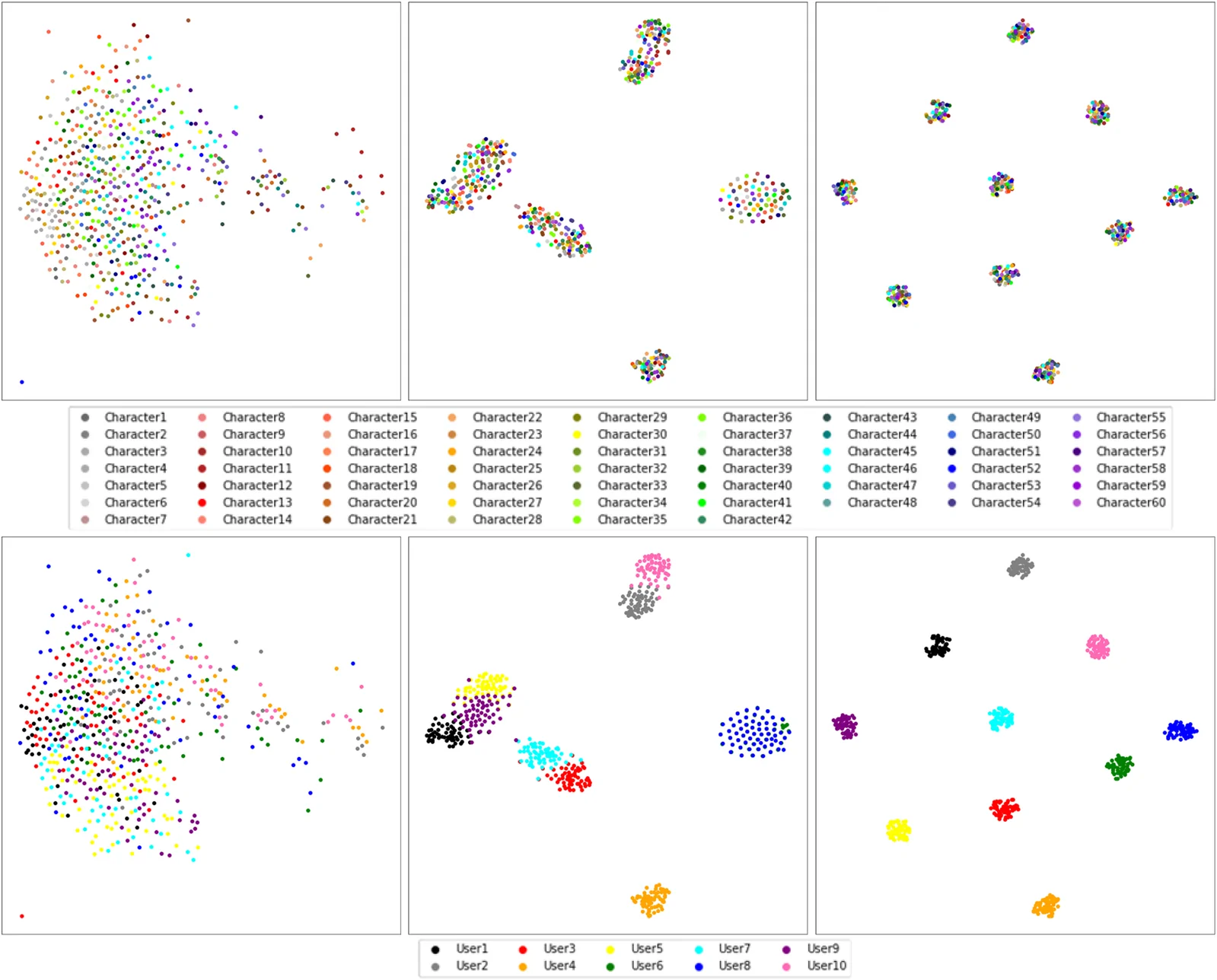
The figure shows the distribution of t-SNE [56] dimensional reduction for character encoding in different scenarios. The first row is the result of clustering by the character with 60 different characters; the second row is the result of clustering by the user with 10 users. The first column is the result of dimensional reduction by the original image information; the second column results from clustering without using one-hot encoding; the third column results from clustering after concatenating the one-hot encoding of characters.

Usually, the handwriting identification task does not limit the characters to be discriminated, and the characters set is large, so it is not easy to use the one-hot encoding to filter the character meaning during the training process. While in the handwriting comparison task, there is a fixed set of characters, which can provide the model with additional character one-hot encoding during the training process, thus making the model focus on the strokes feature extraction of signature image and not suitable for the other datasets. The downstream task in this paper is to prevent surrogate exam-takers, where a test is divided into multiple exams, each of which requires the candidate to write the same paragraph of text, and then determine whether the same person writes the character on different examination papers. We use a single character to discriminate against the user. We can stack the multiple character result to improve the discriminative accuracy in the downstream task. In previous work, traditional machine learning methods require much work for image prepossessing, which is fast but not highly scalable for verifying signatures; deep learning networks can not only automatically extract features but also have better robustness to panning and blocking of signature images, and the additional one-hot encoding can prompt the model to remove the information of the character from the feature extraction process. Fig 7 has two meanings that show the strokes feature information is essential in the evaluation process. First, comparing the images in column 1 with those in columns 2 and 3, we can see that the dimensional reduction of character images are randomly distributed, and the siamese network can classify the original images; second, comparing the images in columns 2 and 3 we can observe that the CNN construct in the siamese network extracts some additional character information without the one-hot encoding of characters, for example, in the second row and second column of the plot, the different user wrote the characters still have less spatially distant due to the same glyphs except user 4; while with the extra one-hot encoding, the siamese network will focus on extracting the handwriting information, thus making the character cluster interval of each user very obvious, so the characters were written by the different user have more significant spatially distant.

**Fig 8:**
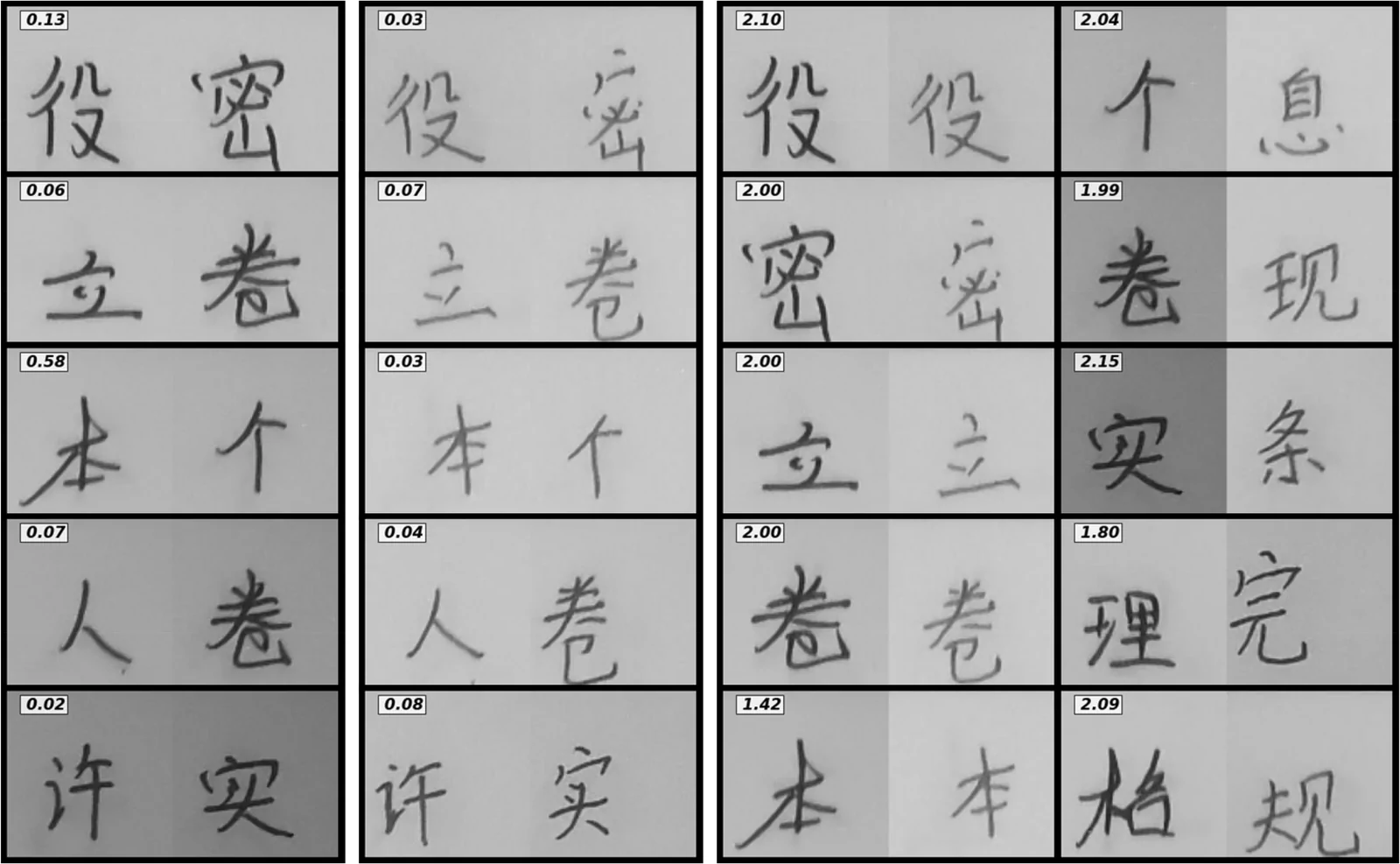
The number in the upper left corner of each image represents the similarity of the sample pairs. The ten sample pairs in the first and second columns are from two different users; the five sample pairs in the third column show the similarity of the same characters from different users, and the five sample pairs in the fourth column show the similarity of different characters from different users. The Chinese characters in each pair are described from left to right. In the first and second columns (from top to bottom): (1) yi (to serve or labor) and mi (secret or dense); (2) li (to stand or establish) and juan (volume or scroll); (3) ben (root or book) and ge (measure word or individual); (4) ren (person or people) and juan (volume or scroll); (5) xu (to allow or permit) and shi (real or true). In the third column, the identical characters written by different users (from top to bottom) are yi, mi, li, juan, and ben, each paired with itself. In the fourth column, the different characters from different users (from top to bottom) are: (1) ge (individual) and xi (breath or rest); (2) juan (volume) and xian (present or to appear); (3) shi (real) and tiao (strip or article); (4) li (reason or principle) and wan (complete or finish); (5) ge (standard or lattice) and gui (rule or regulation).

The specific similarity distance results of positive and negative paired data in Fig 8 show that the same users all have low similarity values for their handwritten characters. In contrast, different users, regardless of whether the characters are the same, have high similarity values. The classification result in the first column of the third row is 0.58, which is slightly larger than the threshold value of 0.5. The characters of the same user are incorrectly discriminated against as characters of different users. This error is that there are fewer character strokes and insufficient handwriting information that can be provided to the model for discriminating.

Training data is crucial for the deep learning models. The character-based signature dataset we collected can be used to obtain balanced training data by randomly sampling positive and negative sample pairs and avoid the long-tail problem. In this paper, we also add dozens of font character images to extend the training data and enhance the model generalization ability.

**Fig 9:**
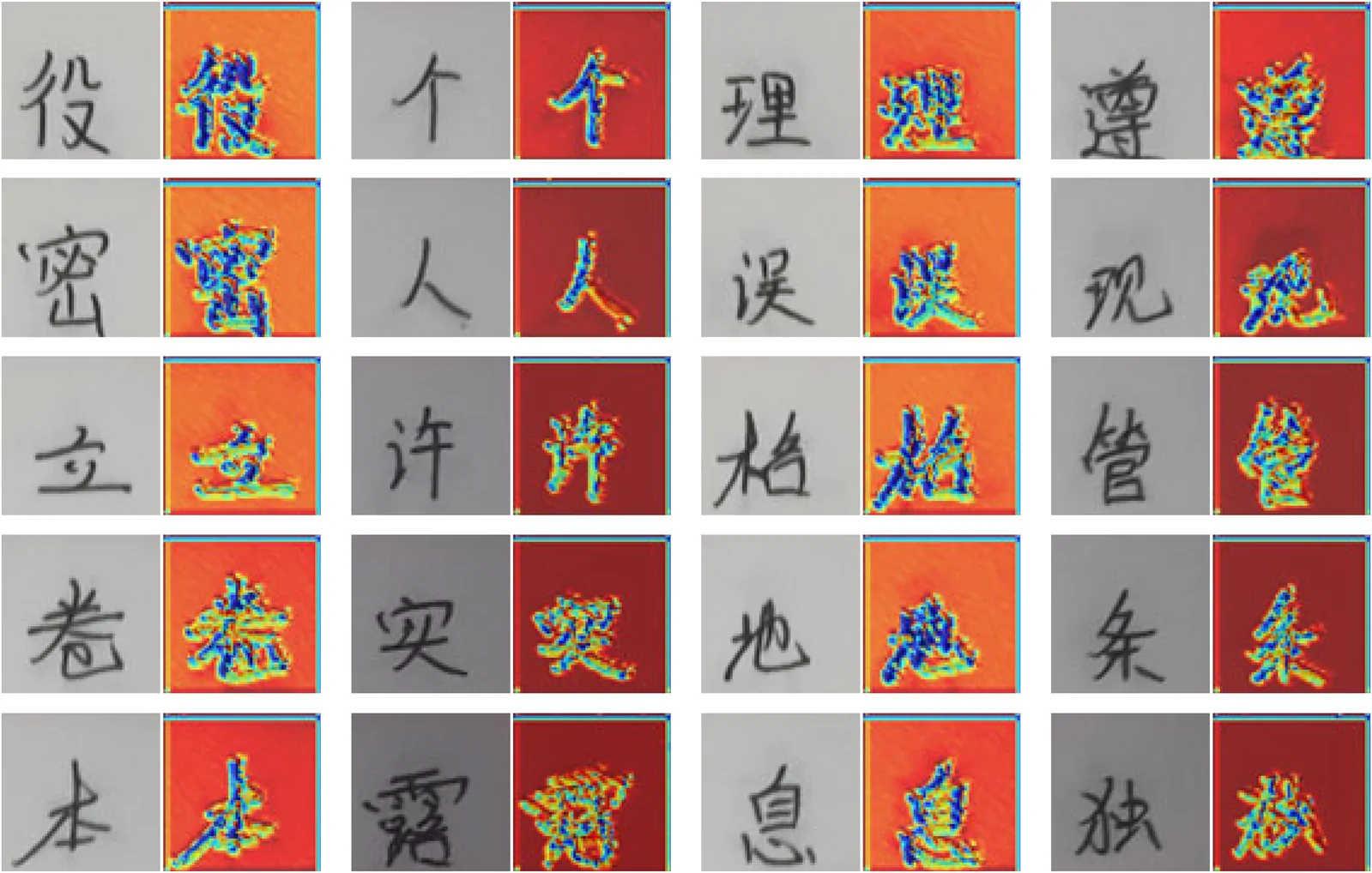
Comparison of the original image with the result of the overlay of the spatial attention heat map, the graph contains the comparison result of 20 characters, the value from small to large corresponds to the color from dark to light. For the original grayscale images (left half of each pair), the characters are described column by column from left to right, and row by row from top to bottom. Column 1: yi (to serve or labor), mi (secret or dense), li (to stand or establish), juan (volume or scroll), and ben (root or book). Column 2: ge (measure word or individual), ren (person or people), xu (to allow or permit), shi (real or true), and lu (dew). Column 3: li (reason or principle), wu (mistake or error), ge (grid or pattern), di (ground or earth), and xi (breath or rest). Column 4: zun (to comply with or observe), xian (to appear or present), guan (to manage or tube), tiao (strip or article), and du (alone or independent).

For the attention mechanism of the CLASN model, we use the spatial attention module in CBAM. Experiments show that channel attention does not significantly improve the effect for grayscale images like signature images. Using only spatial attention can make the model discriminate better for Chinese characters with simple strokes on the one hand and improve its computational efficiency on the other. The visualization of the attention matrix is usually used in NLP, which can visualize the attention weight between tokens; for example, in reading comprehension tasks, the visualization of the attention matrix can represent the article-question attention in different colors. The research in this paper belongs to the CV domain. It uses the spatial attention mechanism, so we observe the model’s attention region to the handwriting through the feature map. The specific results are shown in Fig 9. We extract the feature map of the spatial attention layer after the second convolutional layer and then convert it into a heat map and put it together with the original character picture. We can notice that the spatial attention layer mainly focuses on the handwriting strokes information in the character picture. The location of the brighter color in the heat map indicates that the region has a higher weight. Since the original character picture’s background is not processed, the model also has different weights for different background colors.

## 5. Conclusion and future work

This paper presents a comprehensive study on character-level attention Siamese networks for Chinese handwriting comparison. Unlike traditional handwriting verification tasks that process entire signatures or multiple characters, our proposed CLASN model operates at the character level, enabling finer-grained analysis with reduced sensitivity to spacing, rotation, and alignment variations.

The key findings of this work can be synthesized as follows: (1) Character-level processing significantly reduces noise sensitivity, with CLASN achieving 88.44% accuracy and CLASNF achieving 90.78% accuracy, representing a 16.12% improvement over the baseline SVM method (81.67% ACC). (2) The spatial attention mechanism successfully focuses on discriminative stroke regions, contributing a 2.52% ACC improvement over the baseline. (3) Character one-hot encoding enables effective feature disentanglement, contributing a 3.09% ACC improvement.

The proposed method has practical implications for examination security, financial document verification, and forensic analysis. The CLASN model achieves state-of-the-art performance with FAR of 11.93%, FRR of 9.36%, and ACC of 90.78%, validating the effectiveness of combining spatial attention with character identity encoding for discriminative handwriting feature extraction. The generalization capability of CLASN is supported by several design choices: the inclusion of 50 font types in training improves cross-domain performance (evidenced by the 2.65% accuracy improvement of CLASNF), suggesting the model learns generic handwriting style features rather than memorizing specific patterns, while character-level processing inherently enhances robustness to spacing and rotation variations. However, real-world deployment involves important trade-offs: the model requires a fixed character set known in advance, making it unsuitable for open-vocabulary handwriting comparison, and the character-level approach necessitates segmenting input into individual characters, which introduces preprocessing overhead. Regarding scalability, the lightweight CNN architecture (5 convolutional + 2 fully connected layers) enables efficient inference (approximately 5ms per character pair on standard GPUs), and expanding the current 60-character dataset to cover 3,500 frequently used Chinese characters would enhance practical applicability without requiring architectural changes.

Future work will focus on expanding CCSD to cover more Chinese characters, exploring triplet network architectures for improved efficiency, and developing end-to-end systems for practical deployment in security applications. The practical deployment of CLASN involves several considerations. For examination security applications, the system can be integrated with existing candidate registration databases, where reference handwriting samples are collected during registration and compared against exam-time samples. The real-time inference capability (enabled by the lightweight CNN architecture) supports processing thousands of comparisons within minutes, making it feasible for large-scale examinations. For financial document verification, the model can be deployed as an auxiliary authentication mechanism alongside traditional signature verification systems. The character-level analysis provides an additional layer of security by examining individual characters within signatures rather than treating the entire signature as a single pattern. In forensic analysis, the quantifiable similarity scores (ranging from 0 to 1) provide objective evidence that can complement expert opinions. The attention heatmaps offer interpretability by highlighting which stroke regions contributed most to the similarity judgment, potentially aiding forensic document examiners in their analysis.

## References

[1] Vaswani A, Shazeer N, Parmar N, Uszkoreit J, Jones L, Gomez AN, et al. Attention is all you need. Proceedings of the 31st International Conference on Neural Information Processing Systems. NIPS’17. Red Hook, NY, USA: Curran Associates Inc.; 2017. p. 6000–10.

[2] Ahmed K, Keskar NS, Socher R. Weighted transformer network for machine translation. arXiv:171102132 [Preprint]. 2017.

[3] Bahdanau D, Cho K, Bengio Y. Neural machine translation by jointly learning to align and translate. 3rd International Conference on Learning Representations, ICLR 2015; 2015.

[4] Dai J, Qi H, Xiong Y, Li Y, Zhang G, Hu H, et al. Deformable convolutional networks. Proceedings of the IEEE International Conference on Computer Vision; 2017. p. 764–73.

[5] Hu J, Shen L, Sun G. Squeeze-and-excitation networks. Proceedings of the IEEE Conference on Computer Vision and Pattern Recognition; 2018. p. 7132–41.

[6] Bello I, Zoph B, Vaswani A, Shlens J, Le QV. Attention augmented convolutional networks. Proceedings of the IEEE International Conference on Computer Vision; 2019. p. 3286–95.

[7] Zhang H, Wu C, Zhang Z, Zhu Y, Zhang Z, Lin H, et al. Resnest: split-attention networks. arXiv:200408955 [Preprint]. 2020.

[8] Huang C, Zhu L. Robust evaluation method of communication network based on the combination of complex network and big data. Neural Comput Appl. 2020;33(3):887–96. doi: 10.1007/s00521-020-05264-0

[9] Partiningsih NDA, Fratama RR, Sari CA, Rachmawanto EH, et al. Handwriting ownership recognition using contrast enhancement and LBP feature extraction based on KNN. 2018 5th International Conference on Information Technology, Computer, and Electrical Engineering (ICITACEE). IEEE; 2018. p. 342–6.

[10] Lingyan W. Research and implement of Chinese OCR system. J Jilin Univ (Inf Sci Ed). 2020;38(2):199–205.

[11] Ilmi N, Budi WTA, Nur RK. Handwriting digit recognition using local binary pattern variance and K-Nearest Neighbor classification. 2016 4th International Conference on Information and Communication Technology (ICoICT); 2016.

[12] Slimane F, Märgner V. A new text-independent GMM writer identification system applied to Arabic handwriting. 2014 14th International Conference on Frontiers in Handwriting Recognition. IEEE; 2014. p. 708–13.

[13] Suresh R, Rajasri S. Forgery detection in academic certificates using hybrid deep learning framework. 2026 7th International Conference on Mobile Computing and Sustainable Informatics (ICMCSI); 2026. 10.1109/icmcsi67283.2026.11412772

[14] Raj A, Khan T. Signature forgery detection using deep learning. IJARSCT. 2025:48–52. doi: 10.48175/ijarsct-30008

[15] Chou J, Yeh C, Lee H, Lee L. Multi-target voice conversion without parallel data by adversarially learning disentangled audio representations. Proc Interspeech 2018; 2018. p. 501–5.

[16] Hsu CC, Hwang HT, Wu YC, Tsao Y, Wang HM. Voice conversion from non-parallel corpora using variational auto-encoder. 2016 Asia-Pacific Signal and Information Processing Association Annual Summit and Conference (APSIPA). IEEE; 2016. p. 1–6.

[17] Song L, Gong D, Li Z, Liu C, Liu W. Occlusion robust face recognition based on mask learning with pairwise differential siamese network. Proceedings of the IEEE International Conference on Computer Vision; 2019. p. 773–82.

[18] Bromley J, Bentz JW, Bottou L, Guyon I, LeCun Y, Moore C, et al. Signature verification using a “siamese” time delay neural network. Int J Patt Recogn Artif Intell. 1993;07(04):669–88. doi: 10.1142/s0218001493000339

[19] Li B, Yan J, Wu W, Zhu Z, Hu X. High performance visual tracking with siamese region proposal network. Proceedings of the IEEE Conference on Computer Vision and Pattern Recognition; 2018. p. 8971–80.

[20] Ichida AY, Meneguzzi F, Ruiz DD. Measuring semantic similarity between sentences using a siamese neural network. 2018 International Joint Conference on Neural Networks (IJCNN). IEEE; 2018. p. 1–7.

[21] Das A, Yenala H, Chinnakotla M, Shrivastava M. Together we stand: siamese networks for similar question retrieval. Proceedings of the 54th Annual Meeting of the Association for Computational Linguistics (Volume 1: Long Papers); 2016. p. 378–87.

[22] Srihari SN, Cha SH, Arora H, Lee S. Individuality of handwriting: a validation study. Proceedings of Sixth International Conference on Document Analysis and Recognition. IEEE; 2001. p. 106–9.

[23] of New York at Buffalo SU. Center of Excellence for Document Analysis and Recognition. [cited 2020 Nov 20]. Available from: https://cedar.buffalo.edu/Databases/

[24] Ortega-Garcia J, Fierrez-Aguilar J, Simon D, Gonzalez J, Faundez-Zanuy M, Espinosa V, et al. MCYT baseline corpus: a bimodal biometric database. IEE Proc Vis Image Process. 2003;150(6):395. doi: 10.1049/ip-vis:20031078

[25] Pal S, Blumenstein M, Pal U. Hindi off-line signature verification. 2012 International Conference on Frontiers in Handwriting Recognition. IEEE; 2012. p. 373–8.

[26] Pal S, Alaei A, Pal U, Blumenstein M. Off-line Bangla signature verification: an empirical study. The 2013 International Joint Conference on Neural Networks (IJCNN). IEEE; 2013. p. 1–7.

[27] Ferrer MA, Diaz-Cabrera M, Morales A. Synthetic off-line signature image generation. 2013 international conference on biometrics (ICB). IEEE; 2013. p. 1–7.

[28] Ferrer MA, Diaz-Cabrera M, Morales A. Static signature synthesis: a neuromotor inspired approach for biometrics. IEEE Trans Pattern Anal Mach Intell. 2015;37(3):667–80. doi: 10.1109/TPAMI.2014.2343981 26353268

[29] Deng W, Yu X, Li H, Du S, He B. Multi‐task learning for chinese character and radical recognition with dynamic channel‐spatial attention and rotational positional encoding. IET Image Process. 2025;19(1). doi: 10.1049/ipr2.70213

[30] Liwicki M, Malik MI, Van Den Heuvel CE, Chen X, Berger C, Stoel R, et al. Signature verification competition for online and offline skilled forgeries (sigcomp2011). 2011 International Conference on Document Analysis and Recognition. IEEE; 2011. p. 1480–4.

[31] Wei P, Li H, Hu P. Inverse discriminative networks for handwritten signature verification. Proceedings of the IEEE Conference on Computer Vision and Pattern Recognition; 2019. p. 5764–72.

[32] Ji X, Sun H, Ning Y, Wu M, Zhang C. GTR: General handwritten lines text recognition dataset. International Conference on Content-Based Multimedia Indexing. 2025. 10.1109/CBMI66578.2025.11339304

[33] Chorowski J, Bahdanau D, Cho K, Bengio Y. End-to-end continuous speech recognition using attention-based recurrent NN: first results. arXiv:14121602 [Preprint]. 2014.

[34] Tay Y, Luu AT, Hui SC. Multi-pointer co-attention networks for recommendation. Proceedings of the 24th ACM SIGKDD International Conference on Knowledge Discovery & Data Mining; 2018. p. 2309–18.

[35] Dhingra B, Liu H, Yang Z, Cohen WW, Salakhutdinov R. Gated-attention readers for text comprehension. arXiv:160601549 [Preprint]. 2016.

[36] Yang Z, Yang D, Dyer C, He X, Smola A, Hovy E. Hierarchical attention networks for document classification. Proceedings of the 2016 Conference of the North American Chapter of the Association for Computational Linguistics: Human Language Technologies; 2016. p. 1480–9.

[37] Zhao B, Wu X, Feng J, Peng Q, Yan S. Diversified visual attention networks for fine-grained object classification. IEEE Trans Multimedia. 2017;19(6):1245–56. doi: 10.1109/tmm.2017.2648498

[38] Mnih V, Heess N, Graves A. Recurrent models of visual attention. Adv Neural Inf Process Syst. 2014;27:2204–12.

[39] Wang F, Jiang M, Qian C, Yang S, Li C, Zhang H, et al. Residual attention network for image classification. Proceedings of the IEEE Conference on Computer Vision and Pattern Recognition; 2017. p. 3156–64.

[40] Du Y, Yuan C, Li B, Zhao L, Li Y, Hu W. Interaction-aware spatio-temporal pyramid attention networks for action classification. Proceedings of the European Conference on Computer Vision (ECCV); 2018. p. 373–89.

[41] Niu Y, Zhang H, Zhang M, Zhang J, Lu Z, Wen JR. Recursive visual attention in visual dialog. Proceedings of the IEEE Conference on Computer Vision and Pattern Recognition; 2019. p. 6679–88.

[42] Shaikh M, Duan T, Chauhan M, Srihari S. Attention based writer independent verification. arXiv (Cornell Univ). 2020:373–9. doi: 10.1109/icfhr2020.2020.00074

[43] Hossain SGS, Ghosh M, Obaidullah SM, Roy K. Writer identification using cross-script signature images. LNNS. 2025:45–55. doi: 10.1007/978-981-96-2700-4_4

[44] Lin CQ, Wang DH, Xiao SX, Chi XK, Wang CM, Zhang XY, et al. Offline handwriting verification based on siamese network and multi-channel fusion. Acta Autom Sin. 2024;50(8):1660–70. doi: 10.16383/j.aas.c230777

[45] Liu R, Shi Y, Tang X, Liu X. Combining multi-scale fusion and attentional mechanisms for assessing writing accuracy. Appl Sci. 2025;15(3):1204. doi: 10.3390/app15031204

[46] Wagh T, Kulkarni S. Handwritten signature verification using transfer learning enhanced siamese neural networks. Int J Eng Res Technol. 2026;15(03). doi: 10.5281/zenodo.19440089

[47] Afzali P, Rezapour A, Jordehi A. Leveraging deep feature learning for handwriting biometric authentication. SHILAP Rev lepidopterol. 2024. doi: 10.22105/riej.2024.432510.1412

[48] Huang FH, Lu HM. Multiscale feature learning using co-tuplet loss for offline handwritten signature verification. arXiv (Cornell Univ). 2023:abs/2308.00428. doi: 10.48550/arxiv.2308.00428

[49] Chicco D. Siamese neural networks: an overview. Artif Neural Netw. 2021;2190:73–94. doi: 10.1007/978-1-0716-0826-5_3 32804361

[50] Hadsell R, Chopra S, LeCun Y. Dimensionality reduction by learning an invariant mapping. 2006 IEEE Computer Society Conference on Computer Vision and Pattern Recognition (CVPR’06). vol. 2. IEEE; 2006. p. 1735–42.

[51] Woo S, Park J, Lee J-Y, Kweon IS. CBAM: Convolutional block attention module. Proceedings of the European Conference on Computer Vision (ECCV); 2018. p. 3–19.

[52] Abdallah A, Hamada M, Nurseitov D. Attention-based fully gated CNN-BGRU for Russian handwritten text. J Imaging. 2020;6(12):141. doi: 10.3390/jimaging6120141 34460538 PMC8321198

[53] Kang L, Toledo JI, Riba P, Villegas M, Fornés A, Rusiñol M. Convolve, attend and spell: an attention-based sequence-to-sequence model for handwritten word recognition. German Conference on Pattern Recognition. Springer; 2019. p. 459–72.

[54] Sharif M, Khan MA, Faisal M, Yasmin M, Fernandes SL. A framework for offline signature verification system: best features selection approach. Pattern Recognit Lett. 2020;139:50–9. doi: 10.1016/j.patrec.2018.01.021

[55] Dinesh Kumar DS, Rao PV. RETRACTED: Implementing and analysing FAR and FRR for face and voice recognition (multimodal) using KNN classifier. Int J Intell Unmanned Syst. 2019;8(1):55–67. doi: 10.1108/ijius-02-2019-0015

[56] Van der Maaten L, Hinton G. Visualizing data using t-SNE. J Mach Learn Res. 2008;9(11).

