# Supplementary material for: Character level attention siamese networks for Chinese handwriting comparison
Source: PLoS One. 2026 Sep 17;21(9):e0353503. doi: 10.1371/journal.pone.0353503 (PMC13585223; doi:10.1371/journal.pone.0353503)
Supplement: Supplementary file 1 [file pone.0353503.s001.docx]

Response to Reviewers' Comments

**Dear Editor and Reviewers,**

Thank you for providing valuable comments and suggestions on our manuscript "Character Level Attention Siamese Networks for Chinese Handwriting Comparison". We have carefully revised the manuscript according to the reviewers' feedback. Below, we provide point-by-point responses to all comments.

Sincerely,
Huaqing Mao and Li Zhu

# Reviewer #1: General Notes

## 1. Figure Positioning

**Comment:** Several figures are not positioned immediately after their first reference in the text, which disrupts the logical flow and does not comply with standard journal formatting practices.

**Response:** Thank you for pointing out this formatting issue. We have thoroughly reviewed the entire manuscript and repositioned all figures to ensure that each figure appears immediately after its first reference in the text. Specifically, we have verified the placement of Figures 1-9 to comply with the journal's latex formatting guidelines.

## 2. Figure Clarity and Visual Quality

**Comment:** Some figures, particularly those illustrating the model and network architecture, lack sufficient clarity and visual quality, making it difficult to fully interpret the proposed methodology.

**Response:** Thank you for the helpful comment regarding figure quality. We have carefully revised Figure 4 (CLASN architecture) by horizontally truncating the original figure to make it more compact and clearer. The revised figure now provides a better visual representation of the proposed architecture while maintaining all essential details including the dual-channel structure, layer dimensions, and data flow. The parameter counts (80/2,336/18,496/73,856/295,168/16,385,000/10,010) remain clearly visible in the caption for reference.

# Abstract Section

## 1. Quantitative Results

**Comment:** The abstract does not report quantitative results (FAR, FRR, ACC), which are essential for assessing model effectiveness at a glance.

**Response:** Thank you for the helpful comment. We have revised the abstract to include key quantitative performance metrics. The revised abstract now explicitly states: "Specifically, the proposed CLASN+Fonts model achieves a False Acceptance Rate (FAR) of 11.93%, False Rejection Rate (FRR) of 9.36%, and Accuracy (ACC) of 90.78%, representing a 44.06% reduction in FAR, 42.6% reduction in FRR, and 16.12% improvement in ACC over the baseline SVM method." This allows readers to quickly assess the effectiveness of the proposed model.

## 2. Conceptual Distinction

**Comment:** The distinction between handwriting comparison, signature verification, and character-level comparison is unclear, leading to conceptual ambiguity.

**Response:** Thank you for pointing out this conceptual ambiguity. In the revised manuscript, we have explicitly clarified the distinctions among these three concepts in the Abstract and Introduction sections. We added: "Unlike signature verification which treats the entire signature as a single biometric pattern, or general handwriting comparison which analyzes text at the word or paragraph level, our approach operates at the character level, enabling finer-grained analysis with reduced sensitivity to spacing and alignment variations." This clarification removes the ambiguity and clearly positions the proposed method.

## 3. Experimental Rigor

**Comment:** The evaluation of the proposed model is described qualitatively, without clear indication of experimental rigor.

**Response:** Thank you for the helpful comment. In the revised manuscript, we have strengthened the evaluation by complementing the qualitative analysis with comprehensive quantitative results. We have added detailed experimental setup information including dataset composition (800 users, 60 characters per user, 48,000 handwritten images), training/validation/test split (600/100/100 users), and data augmentation details (positive-negative pair generation with N_pos = 60,000 and N_neg = 90,000). The results section now includes comprehensive comparison with 4 baseline methods (SVM, CSN, SCSN, CLASN) with detailed FAR, FRR, and ACC metrics for all methods.

# Introduction Section

## 1. Research Problem Introduction

**Comment:** The main research problem is not clearly introduced at the beginning of the section.

**Response:** Thank you for the helpful comment. In the revised manuscript, we have explicitly introduced the main research problem at the beginning of the Introduction section: "Handwriting comparison is a critical task in forensic analysis, financial verification, and examination security. The core challenge is to reliably determine whether two handwriting samples originate from the same individual. While existing methods have achieved notable success in signature verification for Latin scripts, Chinese handwriting comparison presents unique challenges due to the complex stroke structures of Chinese characters and the lack of specialized datasets and methods designed for character-level analysis." This clearly motivates the subsequent methodology and analysis.

## 2. Research Question and Hypothesis

**Comment:** The introduction lacks a clearly formulated research question or hypothesis.

**Response:** Thank you for the helpful comment. In the revised manuscript, we have explicitly formulated the main research question and hypothesis. The Research Question is: "Can a character-level attention-based Siamese network effectively capture discriminative stroke features for Chinese handwriting comparison, and will incorporating character identity information through one-hot encoding improve the model's ability to focus on handwriting style rather than character structure?" The Research Hypothesis states: "(1) Processing handwriting at the character level (rather than whole signature) will reduce sensitivity to noise factors such as character spacing and rotation. (2) Incorporating spatial attention mechanisms will enable the model to focus on the most discriminative stroke regions. (3) Providing character identity through one-hot encoding will help disentangle handwriting style features from character structure features, leading to more accurate comparison."

## 3. Literature Overview

**Comment:** The literature overview is insufficient, with only a small number of references and no structured discussion of prior work.

**Response:** Thank you for the helpful comment. In the revised manuscript, we have substantially expanded the literature overview by adding 15+ additional references from 2019-2025, particularly focusing on recent advances in Siamese networks for writer verification, attention mechanisms in handwriting recognition, and deep learning approaches for signature verification. We have reorganized the Related Work section into three structured subsections: Traditional machine learning methods, Deep learning methods, and Attention mechanisms. For each category, we now discuss representative methods, their strengths and limitations, and relevance to our proposed approach.

## 4. Research Gap Identification

**Comment:** There is no explicit identification of a research gap that directly motivates the proposed method.

**Response:** Thank you for the helpful comment. In the revised manuscript, we have explicitly identified four critical research gaps: (1) Dataset Gap: Existing public datasets focus primarily on Latin script signatures at the whole-signature level, lacking large-scale Chinese character-level datasets. (2) Methodological Gap: Current approaches process entire signatures, making them susceptible to noise from character spacing and rotation. (3) Feature Disentanglement Gap: Existing methods struggle to separate handwriting style from character structure features. (4) Attention Application Gap: Attention mechanisms are underexplored for character-level handwriting comparison. Our proposed CLASN method directly addresses these gaps.

## 5. Contributions Derivation

**Comment:** Contributions are listed, but they are not clearly derived from limitations in existing literature.

**Response:** Thank you for the helpful comment. In the revised manuscript, we have clarified the relationship between identified limitations and contributions: (1) Dataset limitation → We build CCSD with 48,000 handwritten character images from 800 users. (2) Noise sensitivity limitation → We propose character-level CLASN processing individual characters. (3) Feature entanglement limitation → We incorporate character one-hot encoding. (4) Attention underexploration limitation → We apply spatial attention mechanisms. This makes the motivation and relevance of each contribution explicit.

# Related Work Section

## 1. Critical Perspective

**Comment:** The section is largely descriptive, resembling a tutorial rather than a critical literature review.

**Response:** Thank you for the helpful comment. We have substantially revised the Related Work section to adopt a more critical and analytical perspective. For each major approach (Traditional Methods, Deep Learning Methods, Siamese Networks, Attention Mechanisms), we now critically assess: (a) What problems does it solve well? (b) What are its fundamental limitations? (c) Under what conditions does it fail? We also analyze how the field has evolved and explicitly discuss why existing methods are insufficient for Chinese character-level comparison. This transforms the section from a tutorial into a critical literature review.

## 2. Comparative Analysis

**Comment:** Prior works are not compared or contrasted in terms of strengths and limitations.

**Response:** We appreciate the reviewer's insightful comment. We have revised the literature review to include explicit comparative analysis. For Traditional Methods: Strengths include computational efficiency and interpretability; Limitations include extensive preprocessing requirements, sensitivity to noise, and poor scalability. For Deep Learning Methods: Strengths include automatic feature learning and robustness; Limitations include data-hunger and feature entanglement issues. For Siamese Networks: Strengths include natural fit for comparison tasks; Limitations include focus on whole signatures rather than character-level. This comparative analysis clearly positions our method relative to prior work.

## 3. Insufficiency Explanation

**Comment:** The discussion does not clearly explain why existing methods are insufficient for the proposed task.

**Response:** Thank you for the helpful comment. We have clarified why existing methods are insufficient for Chinese character-level handwriting comparison. Traditional methods like KNN and SVM are sensitive to character rotation, variable spacing, and background noise—factors common in real-world Chinese handwriting. Standard CNNs process entire signatures, learning both handwriting style AND character structure simultaneously, leading to confusion when comparing characters with similar structures but different handwriting styles. Current Siamese networks are designed for Latin script signatures and lack mechanisms to explicitly handle character identity, making them not directly applicable to Chinese character-level comparison.

## 4. Connection to Proposed Approach

**Comment:** The connection between reviewed studies and the proposed approach is weakly articulated.

**Response:** Thank you for the helpful comment. We have strengthened the connection between reviewed studies and our proposed approach. Building on the observation that traditional methods suffer from limited feature learning, we adopt CNN-based feature extraction with Siamese architecture. While Siamese networks have been successfully applied to signature verification, we extend this approach to the character level, motivated by the need to reduce noise sensitivity. Inspired by feature disentanglement approaches, we explicitly provide character identity through one-hot encoding to address the limitation of standard CNNs that learn both style and structure simultaneously.

## 5. Missing References

**Comment:** Some key references relevant to attention-based similarity learning and writer verification are missing.

**Response:** Thank you for the helpful comment. We have added several key references including: (1) Attention-Based Similarity Learning: Shaikh et al. (2020),Hossain et al. (2025) (2) Writer Verification: Suresh et al. (2026), Raj et al. (2025); (3) Chinese Handwriting Analysis: Deng et al. (2025), Ji et al. (2022), Wang et al. (2023). These references provide a more complete and balanced background.

# Attention Siamese Networks Section

## 1. Methodological Motivation

**Comment:** Although technically detailed, the section lacks a high-level methodological motivation explaining why specific architectural choices were made.

**Response:** We have added high-level methodological motivation for key architectural choices: (1) Siamese Architecture: Chosen because handwriting comparison is fundamentally a similarity learning problem; shared weights ensure comparable embeddings. (2) Character-Level Processing: Motivated by the observation that whole-signature methods suffer from noise in spacing, rotation, and alignment. (3) Spatial Attention: Chosen over channel attention because character images are grayscale (single channel), making channel attention less informative. (4) One-Hot Encoding: Inspired by feature disentanglement research to filter out character structure information and focus on handwriting style features.

## 2. Implementation Details

**Comment:** The section is overly implementation-focused, with excessive low-level details that could be moved to an appendix.

**Response:** Thank you for this valuable suggestion. We have restructured the experimental sections to improve conciseness and logical flow. Specifically, we have merged the original Section 4.3 (Implementation Details) into Section 4.2, creating a new unified section titled "Evaluation Metrics and Experimental Setup". This integration offers several advantages:

Enhanced Logical Coherence: The progression from evaluation criteria (metrics definition) to experimental validation (training setup) creates a seamless narrative flow for understanding our experimental framework.

Selective Detail Preservation: We have removed trivial hyperparameter specifications (e.g., exact learning rate, batch sizes, image dimensions) that represent standard deep learning practices, while retaining task-specific strategies critical for reproducibility, such as the 1.5:1 negative-positive sampling ratio and the contrastive loss margin (r=2.0 ).

Eliminated Redundancy: By consolidating these complementary aspects of the experimental design into one section, we avoid the fragmentation of information while maintaining sufficient detail for readers to understand and reproduce our key results.

# Experiments Section

## 1. Additional Metrics (ROC/EER)

**Comment:** No additional critical metrics (e.g., ROC/EER) are discussed, which could further strengthen evaluation.

**Response:** Thank you for the helpful suggestion regarding additional evaluation metrics. We acknowledge that ROC curves and EER (Equal Error Rate) are valuable metrics in biometric verification systems. However, we would like to clarify the following points regarding our evaluation methodology:

(1) Standard Metrics in Handwriting Comparison Domain

In the handwriting comparison and signature verification domain, FAR (False Acceptance Rate), FRR (False Rejection Rate), and ACC (Accuracy) are the standard evaluation metrics widely adopted in the literature. These three metrics comprehensively reflect the model's performance on both positive and negative samples. Specifically:

- FAR measures the likelihood that the model incorrectly accepts an impostor as normal data, corresponding to False Positive Rate (FPR) in ROC analysis.

- FRR measures the likelihood that the model incorrectly rejects normal data, where (1-FRR) corresponds to True Positive Rate (TPR) in ROC analysis.

- ACC provides an overall measure of correct classifications.

(2) Relationship Between FAR/FRR and ROC/EER

The trade-off between FAR and FRR inherently captures the same information as ROC curves. By reporting FAR and FRR values at the operating threshold (margin r = 2.0 in our contrastive loss), we provide sufficient information to understand the model's discrimination capability. The EER (Equal Error Rate) can be estimated from our reported results:

- For CLASN: FAR = 12.72%, FRR = 10.17%, approximate EER ≈ 11.4%

- For CLASNF: FAR = 11.93%, FRR = 9.36%, approximate EER ≈ 10.6%

These EER values are competitive with recent studies. For example, Kulkarni & Wagh (2026) reported EER of 10.08% on signature verification using Siamese+MobileNetV2, while our CLASNF achieves comparable performance (~10.6% EER) on the more challenging character-level comparison task.

(3) Practical Considerations

In real-world deployment scenarios (e.g., examination security systems), the operating threshold is typically adjusted based on the relative cost of false acceptances versus false rejections. Our reported FAR/FRR values at the optimized threshold provide practical guidance for system deployment, whereas EER represents only a single point where the two error rates are equal.

We have added a discussion in Section 4.2 (Evaluation Metrics and Experimental Setup) clarifying the relationship between our reported metrics and standard biometric evaluation measures.

## 2. Ablation Study

**Comment:** The contribution of each component is not clearly demonstrated.

**Response:** Thank you for the helpful comment. We have added an ablation study table showing the contribution of each component: Baseline (no attention, no encoding): FAR 18.42%, FRR 14.23%, ACC 83.67%; + Spatial Attention: FAR 15.18%, FRR 12.45%, ACC 86.19% (improvement of 2.52% ACC); + Character Encoding: FAR 14.67%, FRR 11.82%, ACC 86.76% (improvement of 0.57% ACC); + Both (CLASN): FAR 12.72%, FRR 10.17%, ACC 88.44%; + Fonts (CLASNF): FAR 11.93%, FRR 9.36%, ACC 90.78% (improvement of 2.34% ACC). This demonstrates that each component contributes meaningfully to the overall performance.

# Results Section

## Comparison with Recent Studies (2023-2025)

**Comment:** No comparison was provided between the findings of this researcher and those of peer-reviewed research papers published during the period 2023-2025.

**Response:** Thank you for the valuable suggestion. We have added comprehensive comparisons with recent studies (2023-2025) in the revised Related Work and Results sections.

# Analysis and Discussion Section

## 1. Redundant Explanations

**Comment:** Some explanations in the Analysis and Discussion section repeat observations already evident from the results table.

**Response:** Thank you for the helpful comment. We have revised the Analysis and Discussion section to focus on interpretation rather than restating numerical results. The revised section now discusses: (a) Why CLASN outperforms baselines through attention mechanisms and character encoding analysis; (b) Feature visualization interpretation from t-SNE plots; (c) Attention heatmap analysis showing focus regions; (d) Error analysis identifying misclassified samples; (e) Generalization discussion for unseen users and characters. This provides deeper insights rather than surface-level observations.

## 2. Connection to Research Motivation

**Comment:** The discussion could more explicitly link empirical findings back to the original research motivation.

**Response:** We appreciate the reviewer's insightful comment. We have revised the Discussion section to explicitly relate findings to research motivation: (1) Character-Level Processing: "Our results confirm the hypothesis that character-level processing reduces noise sensitivity. The model achieves consistent performance across varying character spacing and rotation, validating our motivation for moving away from whole-signature approaches." (2) Attention Mechanism: "The attention heatmaps show that the model focuses on stroke regions, confirming that spatial attention successfully highlights discriminative features, validating our hypothesis." (3) Feature Disentanglement: "The t-SNE visualization demonstrates clear clustering by user when character encoding is used, supporting our hypothesis that explicit character identity helps separate style from structure."

# Conclusion Section

## 1. Synthesis Rather Than Repetition

**Comment:** The conclusion largely repeats results rather than synthesizing insights or implications.

**Response:**

Thank you for the valuable comment. We have substantially revised the Conclusion section to synthesize key insights rather than merely repeating numerical results. Specifically:

(1) We restructured the Conclusion to begin with a synthesis paragraph that organizes our findings into three key contributions: (a) character-level processing reduces noise sensitivity, validated by the 16.12% accuracy improvement over baseline; (b) spatial attention mechanisms successfully focus on discriminative stroke regions, contributing 2.52% accuracy gain; and (c) character one-hot encoding enables effective feature disentanglement, contributing 3.09% accuracy improvement.

(2) We added an interpretation of why these components work together: the spatial attention highlights informative regions while character encoding filters out structural information, allowing the model to focus exclusively on handwriting style features.

(3) We connected these findings back to our original research hypotheses stated in the Introduction, demonstrating how our experimental results validate the theoretical motivations.

## 2. Broader Impact and Applicability

**Comment:** Broader impact and applicability of the proposed method are not sufficiently discussed.

**Response:**

Thank you for the helpful suggestion. We have expanded the discussion of broader impact and applicability in the Conclusion section. Specifically, we added a new paragraph discussing practical deployment considerations for each application domain:

(1) For examination security, we describe how CLASN can integrate with existing registration systems and support real-time processing of large-scale examinations.

(2) For financial verification, we explain how the model serves as an auxiliary authentication mechanism that provides additional security through character-level analysis.

(3) For forensic analysis, we highlight the value of quantifiable similarity scores and interpretable attention heatmaps as objective evidence.

## 3. Generalization and Deployment

**Comment:** The section would benefit from a clearer reflection on generalization, scalability, and real-world deployment.

**Response:**

Thank you for the insightful comment. We have added a dedicated section in the Conclusion addressing generalization, scalability, and deployment considerations:

(1) Generalization: We discuss how font augmentation improves cross-domain performance and how character-level processing inherently enhances robustness to spacing and rotation variations.

(2) Scalability: We analyze both dataset expansion potential (from 60 to 3,500 characters) and computational efficiency (~5ms per comparison on standard GPU).

(3) Deployment Limitations: We acknowledge practical constraints including the fixed character set requirement and character segmentation overhead, providing a balanced perspective for potential adopters.

# Reviewer #2 Comments

## 1. Numerical Values in Abstract

**Comment:** The abstract did not specify numerical values for the approved metrics that demonstrate the efficiency of the proposed system.

**Response:** (Combined with Reviewer #1 Abstract comment - same response) We have revised the abstract to include: "The proposed CLASN+Fonts model achieves a False Acceptance Rate (FAR) of 11.93%, False Rejection Rate (FRR) of 9.36%, and Accuracy (ACC) of 90.78%, representing a 44.06% reduction in FAR, 42.6% reduction in FRR, and 16.12% improvement in ACC over the baseline SVM method."

## 2. Related Work Summary Table

**Comment:** The research review of related works was presented without any explanation of the research papers, the data sets used, the numerical values of the measures obtained by each researcher, or the disadvantages of each method in each study.

**Response:** Thank you for the helpful comment. We have added a comprehensive summary table in the Related Work section comparing representative prior studies in terms of methodology, datasets, evaluation metrics, reported performance, and key limitations. The table includes: Srihari et al. [22] (KNN, CEDAR, FAR/FRR 12.4%/18.7%, limitation: requires extensive preprocessing); Ortega-Garcia et al. [24] (GMM, MCYT, EER 8.2%, limitation: limited to whole signatures); Pal et al. [25] (CNN, BHSig-260, ACC 84.3%, limitation: no attention mechanism); Ferrer et al. [27] (HMM, GPDS, FAR/FRR 9.8%/15.2%, limitation: computationally expensive); Wei et al. [30] (IDN, Self-collected, ACC 87.6%, limitation: multi-character processing); Our Work (CLASN, CCSD, FAR/FRR/ACC 11.93%/9.36%/90.78%). This table enables readers to quickly compare methods and understand the landscape.

## 3. Comparison with Recent Studies (2023-2025)

**Comment:** No comparison was provided between the findings of this researcher and those of peer-reviewed research papers published during the period 2023-2025.

**Response:** Thank you for the valuable suggestion. We have added comprehensive comparisons with recent studies (2023-2025) in the revised Related Work and Results sections.

## 4. Additional Diagrams and Tables

**Comment:** Sufficient diagrams and numerical tables were not included to illustrate the steps involved in the processing operation.

**Response:** Thank you for the suggestion regarding additional diagrams. After careful consideration, we believe that the current manuscript already includes sufficient visual elements to illustrate the methodology: Figure 3 provides an overview of the proposed method, Figure 4 illustrates the CLASN architecture, Figure 5 details the spatial attention operation, Figure 6 shows dataset examples, Figure 7 presents t-SNE visualizations, Figure 8 demonstrates similarity results, and Figure 9 displays attention heatmaps. Additionally, Table 1 provides dataset statistics and Table 2 presents comprehensive performance comparisons including ablation studies.

Adding more diagrams would increase the manuscript length without providing substantial additional clarity, as the existing figures already cover the data preprocessing pipeline (Figure 3), model architecture (Figure 4), and attention mechanisms (Figure 5, 9). We have instead enhanced the textual descriptions in Sections 3.2 and 4.1 to provide clearer explanations of the processing steps. If the reviewer feels specific diagrams are essential, we would be happy to add them in the revision.

## 5. Recent References (2022-2025)

**Comment:** The number of published references between 2022-2025 is only one, which is a very small number. Therefore, please double this number to at least two research papers per year during the aforementioned period.

**Response:** Thank you for the valuable suggestion. We have added comprehensive comparisons with recent studies (2023-2025) in the revised Related Work and Results sections. Key recent works and their relationship to our proposed method are summarized below:

(1) Chao-Qun et al. (2024) - Closest Related Work

Chao-Qun et al. proposed an offline handwriting verification method based on Siamese networks with multi-channel fusion, published in Acta Automatica Sinica (2024). Their dual-channel architecture shares similarities with our CLASN, but our approach incorporates several distinct advantages:

- We integrate spatial attention mechanisms (CBAM) to highlight discriminative	stroke regions, while their method uses multi-channel fusion without explicit	attention modeling.

- We incorporate character one-hot encoding for feature disentanglement, which is not addressed in their work.

- Our method is specifically designed for character-level comparison, enabling finer-grained analysis.

(2) Liu et al. (2025) [10] - Attention Mechanisms for Chinese Characters

Liu et al. developed a multi-scale SE (Squeeze-and-Excitation) attention model for handwritten Chinese character recognition, achieving 98.6% accuracy on writing grade examination datasets (Applied Sciences, 2025). While their recognition task differs from our comparison task, their attention mechanism design validates the effectiveness of attention-based approaches for Chinese character analysis. Our spatial attention mechanism complements their channel attention approach, as grayscale character images benefit more from spatial attention than channel attention.

(3) Kulkarni & Wagh (2026) - Siamese Networks for Signature Verification

Kulkarni & Wagh proposed a Transfer Learning-enhanced Siamese Neural Network for signature verification (IJERT, 2026), achieving:

- Accuracy: 90.10%

- FAR: 11.69%

- FRR: 8.10%

- EER: 10.08%

Our CLASNF model achieves comparable performance (ACC: 90.78%, FAR: 11.93%, FRR: 9.36%, estimated EER: ~10.6%) on the more challenging character-level comparison task, where the model must handle 60 different character structures while distinguishing handwriting styles.

(4) Afzali et al. (2024) - Writer Verification with Siamese Networks

Afzali et al. developed a Customized Siamese CNN (CSCNN) for offline writer verification using combined loss functions (Int. J. Research in Industrial Engineering, 2024). Their work demonstrates the effectiveness of Siamese architectures for handwriting biometric authentication across different languages (English and Arabic). Our work extends this approach to Chinese character-level comparison with attention mechanisms and character encoding.

(5) Huang et al. (2023) - Multi-Scale Signature Verification

Huang et al. proposed MS-SigNet with co-tuplet loss for offline handwritten signature verification (arXiv, 2023) and introduced HanSig, a large-scale Chinese signature dataset. While their work focuses on signature-level verification, our CCSD dataset is the first large-scale dataset specifically designed for character-level Chinese handwriting comparison.

## 6. Numerical Values in Conclusion

**Comment:** The conclusions are devoid of any numerical values for the results.

**Response:**

Thank you for pointing out the omission of numerical values in the Conclusion. We have revised the Conclusion section to include specific quantitative results:

CLASN achieves 88.44% accuracy, CLASNF achieves 90.78% accuracy with FAR of 11.93% and FRR of 9.36%, representing a 16.12% improvement over the baseline SVM method. We have also included the contributions of individual components: spatial attention contributes 2.52% ACC improvement, and character encoding contributes 3.09% ACC improvement.
