# Supplementary material for: Character level attention siamese networks for Chinese handwriting comparison
Source: PLoS One. 2026 Sep 17;21(9):e0353503. doi: 10.1371/journal.pone.0353503 (PMC13585223; doi:10.1371/journal.pone.0353503)
Supplement: Peer review history

# Peer review: Decision Letter 0

**DOI:** 10.1371/journal.pone.0353503.r001

**Authors:** Dhamyaa Abbas Nasrawi

26 Jan 2026

Dear Dr. Zhu,

Thank you for submitting your manuscript to PLOS ONE. After careful consideration, we feel that it has merit but does not fully meet PLOS ONE’s publication criteria as it currently stands. Therefore, we invite you to submit a revised version of the manuscript that addresses the points raised during the review process.

==============================

**ACADEMIC EDITOR:**

The manuscript addresses an interesting topic; however, we noticed that approximately 88% of the cited references predate 2020.

In the rapidly evolving fields of Computer Science and Information Technology, maintaining the "currency" of references is not merely a formal requirement but a necessity. The current references strongly suggest that the research does not reflect the latest advancements or the current State-of-the-Art (SOTA). As it stands, the manuscript appears to have been authored several years ago and lacks a connection to contemporary technological trends and recent scholarly breakthroughs.

You are required to integrate significant developments and publications from the last 3–5 years to ensure the work is grounded in the current scientific landscape.

Important Note: Failure to comprehensively address this issue and to provide an updated, relevant bibliography will, unfortunately, lead to the rejection of your manuscript.

We look forward to receiving your revised manuscript.

Best regards,

==============================

Please submit your revised manuscript by Mar 12 2026 11:59PM. If you will need more time than this to complete your revisions, please reply to this message or contact the journal office at plosone@plos.org. When you're ready to submit your revision, log on to https://www.editorialmanager.com/pone/ and select the 'Submissions Needing Revision' folder to locate your manuscript file.

- A letter that responds to each point raised by the academic editor and reviewer(s). You should upload this letter as a separate file labeled 'Response to Reviewers'.
- A marked-up copy of your manuscript that highlights changes made to the original version. You should upload this as a separate file labeled 'Revised Manuscript with Track Changes'.
- An unmarked version of your revised paper without tracked changes. You should upload this as a separate file labeled 'Manuscript'.

If you would like to make changes to your financial disclosure, please include your updated statement in your cover letter. Guidelines for resubmitting your figure files are available below the reviewer comments at the end of this letter.

If applicable, we recommend that you deposit your laboratory protocols in protocols.io to enhance the reproducibility of your results. Protocols.io assigns your protocol its own identifier (DOI) so that it can be cited independently in the future. For instructions see: https://journals.plos.org/plosone/s/submission-guidelines#loc-laboratory-protocols. Additionally, PLOS ONE offers an option for publishing peer-reviewed Lab Protocol articles, which describe protocols hosted on protocols.io. Read more information on sharing protocols at https://plos.org/protocols?utm_medium=editorial-email&utm_source=authorletters&utm_campaign=protocols.

We look forward to receiving your revised manuscript.

Kind regards,

Dhamyaa Abbas Nasrawi, Ph.D.

Academic Editor

PLOS One

Journal Requirements:

When submitting your revision, we need you to address these additional requirements.

1. Please ensure that your manuscript meets PLOS ONE's style requirements, including those for file naming. The PLOS ONE style templates can be found at

https://journals.plos.org/plosone/s/file?id=wjVg/PLOSOne_formatting_sample_main_body.pdf and

https://journals.plos.org/plosone/s/file?id=ba62/PLOSOne_formatting_sample_title_authors_affiliations.pdf

2. Please note that PLOS One has specific guidelines on code sharing for submissions in which author-generated code underpins the findings in the manuscript. In these cases, we expect all author-generated code to be made available without restrictions upon publication of the work. Please review our guidelines at https://journals.plos.org/plosone/s/materials-and-software-sharing#loc-sharing-code and ensure that your code is shared in a way that follows best practice and facilitates reproducibility and reuse.

3. Please provide additional details regarding participant consent. In the ethics statement in the Methods and online submission information, please ensure that you have specified (1) whether consent was informed and (2) what type you obtained (for instance, written or verbal, and if verbal, how it was documented and witnessed). If your study included minors, state whether you obtained consent from parents or guardians. If the need for consent was waived by the ethics committee, please include this information.

If you are reporting a retrospective study of medical records or archived samples, please ensure that you have discussed whether all data were fully anonymized before you accessed them and/or whether the IRB or ethics committee waived the requirement for informed consent. If patients provided informed written consent to have data from their medical records used in research, please include this information.

4. We note that your Data Availability Statement is currently as follows: All relevant data are within the manuscript and its Supporting Information files.

Please confirm at this time whether or not your submission contains all raw data required to replicate the results of your study. Authors must share the “minimal data set” for their submission. PLOS defines the minimal data set to consist of the data required to replicate all study findings reported in the article, as well as related metadata and methods (https://journals.plos.org/plosone/s/data-availability#loc-minimal-data-set-definition).

For example, authors should submit the following data:

- The values behind the means, standard deviations and other measures reported;

- The values used to build graphs;

- The points extracted from images for analysis.

Authors do not need to submit their entire data set if only a portion of the data was used in the reported study.

If your submission does not contain these data, please either upload them as Supporting Information files or deposit them to a stable, public repository and provide us with the relevant URLs, DOIs, or accession numbers. For a list of recommended repositories, please see https://journals.plos.org/plosone/s/recommended-repositories.

If there are ethical or legal restrictions on sharing a de-identified data set, please explain them in detail (e.g., data contain potentially sensitive information, data are owned by a third-party organization, etc.) and who has imposed them (e.g., an ethics committee). Please also provide contact information for a data access committee, ethics committee, or other institutional body to which data requests may be sent. If data are owned by a third party, please indicate how others may request data access.

5. When completing the data availability statement of the submission form, you indicated that you will make your data available on acceptance. We strongly recommend all authors decide on a data sharing plan before acceptance, as the process can be lengthy and hold up publication timelines. Please note that, though access restrictions are acceptable now, your entire data will need to be made freely accessible if your manuscript is accepted for publication. This policy applies to all data except where public deposition would breach compliance with the protocol approved by your research ethics board. If you are unable to adhere to our open data policy, please kindly revise your statement to explain your reasoning and we will seek the editor's input on an exemption. Please be assured that, once you have provided your new statement, the assessment of your exemption will not hold up the peer review process.

6. Please amend the manuscript submission data (via Edit Submission) to include authors Long Tang and Huaqing Mao.

7. If the reviewer comments include a recommendation to cite specific previously published works, please review and evaluate these publications to determine whether they are relevant and should be cited. There is no requirement to cite these works unless the editor has indicated otherwise.

[Note: HTML markup is below. Please do not edit.]

Reviewers' comments:

Reviewer's Responses to Questions

**Comments to the Author**

1. Is the manuscript technically sound, and do the data support the conclusions?

Reviewer #1: Partly

Reviewer #2: Yes

**********

2. Has the statistical analysis been performed appropriately and rigorously?

Reviewer #1: Yes

Reviewer #2: Yes

**********

3. Have the authors made all data underlying the findings in their manuscript fully available?

The PLOS Data policy

Reviewer #1: No

Reviewer #2: Yes

**********

4. Is the manuscript presented in an intelligible fashion and written in standard English?

Reviewer #1: Yes

Reviewer #2: Yes

**********

Reviewer #1: General Notes

1. Several figures are not positioned immediately after their first reference in the text, which disrupts the logical flow and does not comply with standard journal formatting practices.

2. Some figures, particularly those illustrating the model and network architecture, lack sufficient clarity and visual quality, making it difficult to fully interpret the proposed methodology.

In the follwoing the notes I found its important to be considered in the manuscript:

Abstract

The abstract addresses a relevant problem in handwriting comparison and proposes a character-level attention-based Siamese CNN, which is an appropriate and potentially effective approach, particularly for Chinese handwriting. The construction of a large-scale handwritten character dataset is a notable contribution. However,

1. The abstract does not report quantitative results ( FAR, FRR, ACC), which are essential for assessing model effectiveness at a glance.

2. The distinction between handwriting comparison, signature verification, and character-level comparison is unclear, leading to conceptual ambiguity.

3. The evaluation of the proposed model is described qualitatively, without clear indication of experimental rigor.

Introduction Section

1. The main research problem is not clearly introduced at the beginning of the section.

2. The introduction lacks a clearly formulated research question or hypothesis.

3. The literature overview is insufficient, with only a small number of references and no structured discussion of prior work.

4. There is no explicit identification of a research gap that directly motivates the proposed method.

5. Contributions are listed, but they are not clearly derived from limitations in existing literature.

Related Work

1. The section is largely descriptive, resembling a tutorial rather than a critical literature review.

2. Prior works are not compared or contrasted in terms of strengths and limitations.

3. The discussion does not clearly explain why existing methods are insufficient for the proposed task.

4. The connection between reviewed studies and the proposed approach is weakly articulated.

5. Some key references relevant to attention-based similarity learning and writer verification are missing.

Attention Siamese Networks

1. Although technically detailed, the section lacks a high-level methodological motivation explaining why specific architectural choices were made.

2. The section is overly implementation-focused, with excessive low-level details that could be moved to an appendix.

3. The figure illustrating the overall proposed method does not appear immediately after it is referenced.

4. The Siamese network architecture figure is missing or misplaced, reducing clarity.

5. The advantages of the proposed architecture over standard Siamese or attention-based models are not explicitly justified.

Experiments and the rest of the manuscript sections

1. The experimental workflow is clearly organized and well explained.

2. Dataset preparation, training protocol, and baselines are described in a logical sequence.

3. No major methodological gaps are observed in the experimental design.

4. The selected metrics (FAR, FRR, ACC) are appropriate and standard for handwriting verification tasks.

5. Metrics are clearly defined and supported by prior literature.

6. No additional critical metrics (e.g., ROC/EER) are discussed, which could further strengthen evaluation.

7. Section 4.3 (“Detail”) primarily contains implementation parameters and should be merged with the experimental setup or renamed “Implementation Details.”

8. Some explanations in the Analysis and Discussion section repeat observations already evident from the results table.

9. The discussion could more explicitly link empirical findings back to the original research motivation.

10. Figures referenced in the discussion are not always placed consistently after citation.

11. The conclusion largely repeats results rather than synthesizing insights or implications.

12. Broader impact and applicability of the proposed method are not sufficiently discussed.

13. The section would benefit from a clearer reflection on generalization, scalability, and real-world deployment.

Reviewer #2: 1- The abstract did not specify numerical values for the approved metrics that demonstrate the efficiency of the proposed system.

2- The research review of related works was presented without any explanation of the research papers, the data sets used, the numerical values of the measures obtained by each researcher, or the disadvantages of each method in each study. All of this could be clarified in a table summarizing all the relevant papers.

3- No comparison was provided between the findings of this researcher and those of peer-reviewed research papers published during the period 2023-2025.

4- Sufficient diagrams and numerical tables were not included to illustrate the steps involved in the processing operation.

5- The number of published references between 2022-2025 is only one, which is a very small number. Therefore, please double this number to at least two research papers per year during the aforementioned period.

6- The conclusions are devoid of any numerical values for the results.

**********

what does this mean?). If published, this will include your full peer review and any attached files.

If you choose “no”, your identity will remain anonymous but your review may still be made public.

**Do you want your identity to be public for this peer review?**  For information about this choice, including consent withdrawal, please see our Privacy Policy

Reviewer #1: **Yes:**  Mais Saad Safoq

Reviewer #2: No

**********

[NOTE: If reviewer comments were submitted as an attachment file, they will be attached to this email and accessible via the submission site. Please log into your account, locate the manuscript record, and check for the action link "View Attachments". If this link does not appear, there are no attachment files.]

To ensure your figures meet our technical requirements, please review our figure guidelines: https://journals.plos.org/plosone/s/figures

You may also use PLOS’s free figure tool, NAAS, to help you prepare publication quality figures: https://journals.plos.org/plosone/s/figures#loc-tools-for-figure-preparation.

NAAS will assess whether your figures meet our technical requirements by comparing each figure against our figure specifications.

# Author comment: Author response to Decision Letter 1

**DOI:** 10.1371/journal.pone.0353503.r002

2 Jun 2026

Reviewer #1: General Notes

1. Figure Positioning

Comment: Several figures are not positioned immediately after their first reference in the text, which disrupts the logical flow and does not comply with standard journal formatting practices.

Response: Thank you for pointing out this formatting issue. We have thoroughly reviewed the entire manuscript and repositioned all figures to ensure that each figure appears immediately after its first reference in the text. Specifically, we have verified the placement of Figures 1-9 to comply with the journal's latex formatting guidelines.

2. Figure Clarity and Visual Quality

Comment: Some figures, particularly those illustrating the model and network architecture, lack sufficient clarity and visual quality, making it difficult to fully interpret the proposed methodology.

Response: Thank you for the helpful comment regarding figure quality. We have carefully revised Figure 4 (CLASN architecture) by horizontally truncating the original figure to make it more compact and clearer. The revised figure now provides a better visual representation of the proposed architecture while maintaining all essential details including the dual-channel structure, layer dimensions, and data flow. The parameter counts (80/2,336/18,496/73,856/295,168/16,385,000/10,010) remain clearly visible in the caption for reference.

Abstract Section

1. Quantitative Results

Comment: The abstract does not report quantitative results (FAR, FRR, ACC), which are essential for assessing model effectiveness at a glance.

Response: Thank you for the helpful comment. We have revised the abstract to include key quantitative performance metrics. The revised abstract now explicitly states: "Specifically, the proposed CLASN+Fonts model achieves a False Acceptance Rate (FAR) of 11.93%, False Rejection Rate (FRR) of 9.36%, and Accuracy (ACC) of 90.78%, representing a 44.06% reduction in FAR, 42.6% reduction in FRR, and 16.12% improvement in ACC over the baseline SVM method." This allows readers to quickly assess the effectiveness of the proposed model.

2. Conceptual Distinction

Comment: The distinction between handwriting comparison, signature verification, and character-level comparison is unclear, leading to conceptual ambiguity.

Response: Thank you for pointing out this conceptual ambiguity. In the revised manuscript, we have explicitly clarified the distinctions among these three concepts in the Abstract and Introduction sections. We added: "Unlike signature verification which treats the entire signature as a single biometric pattern, or general handwriting comparison which analyzes text at the word or paragraph level, our approach operates at the character level, enabling finer-grained analysis with reduced sensitivity to spacing and alignment variations." This clarification removes the ambiguity and clearly positions the proposed method.

3. Experimental Rigor

Comment: The evaluation of the proposed model is described qualitatively, without clear indication of experimental rigor.

Response: Thank you for the helpful comment. In the revised manuscript, we have strengthened the evaluation by complementing the qualitative analysis with comprehensive quantitative results. We have added detailed experimental setup information including dataset composition (800 users, 60 characters per user, 48,000 handwritten images), training/validation/test split (600/100/100 users), and data augmentation details (positive-negative pair generation with N_pos = 60,000 and N_neg = 90,000). The results section now includes comprehensive comparison with 4 baseline methods (SVM, CSN, SCSN, CLASN) with detailed FAR, FRR, and ACC metrics for all methods.

Introduction Section

1. Research Problem Introduction

Comment: The main research problem is not clearly introduced at the beginning of the section.

Response: Thank you for the helpful comment. In the revised manuscript, we have explicitly introduced the main research problem at the beginning of the Introduction section: "Handwriting comparison is a critical task in forensic analysis, financial verification, and examination security. The core challenge is to reliably determine whether two handwriting samples originate from the same individual. While existing methods have achieved notable success in signature verification for Latin scripts, Chinese handwriting comparison presents unique challenges due to the complex stroke structures of Chinese characters and the lack of specialized datasets and methods designed for character-level analysis." This clearly motivates the subsequent methodology and analysis.

2. Research Question and Hypothesis

Comment: The introduction lacks a clearly formulated research question or hypothesis.

Response: Thank you for the helpful comment. In the revised manuscript, we have explicitly formulated the main research question and hypothesis. The Research Question is: "Can a character-level attention-based Siamese network effectively capture discriminative stroke features for Chinese handwriting comparison, and will incorporating character identity information through one-hot encoding improve the model's ability to focus on handwriting style rather than character structure?" The Research Hypothesis states: "(1) Processing handwriting at the character level (rather than whole signature) will reduce sensitivity to noise factors such as character spacing and rotation. (2) Incorporating spatial attention mechanisms will enable the model to focus on the most discriminative stroke regions. (3) Providing character identity through one-hot encoding will help disentangle handwriting style features from character structure features, leading to more accurate comparison."

3. Literature Overview

Comment: The literature overview is insufficient, with only a small number of references and no structured discussion of prior work.

Response: Thank you for the helpful comment. In the revised manuscript, we have substantially expanded the literature overview by adding 15+ additional references from 2019-2025, particularly focusing on recent advances in Siamese networks for writer verification, attention mechanisms in handwriting recognition, and deep learning approaches for signature verification. We have reorganized the Related Work section into three structured subsections: Traditional machine learning methods, Deep learning methods, and Attention mechanisms. For each category, we now discuss representative methods, their strengths and limitations, and relevance to our proposed approach.

4. Research Gap Identification

Comment: There is no explicit identification of a research gap that directly motivates the proposed method.

Response: Thank you for the helpful comment. In the revised manuscript, we have explicitly identified four critical research gaps: (1) Dataset Gap: Existing public datasets focus primarily on Latin script signatures at the whole-signature level, lacking large-scale Chinese character-level datasets. (2) Methodological Gap: Current approaches process entire signatures, making them susceptible to noise from character spacing and rotation. (3) Feature Disentanglement Gap: Existing methods struggle to separate handwriting style from character structure features. (4) Attention Application Gap: Attention mechanisms are underexplored for character-level handwriting comparison. Our proposed CLASN method directly addresses these gaps.

5. Contributions Derivation

Comment: Contributions are listed, but they are not clearly derived from limitations in existing literature.

Response: Thank you for the helpful comment. In the revised manuscript, we have clarified the relationship between identified limitations and contributions: (1) Dataset limitation → We build CCSD with 48,000 handwritten character images from 800 users. (2) Noise sensitivity limitation → We propose character-level CLASN processing individual characters. (3) Feature entanglement limitation → We incorporate character one-hot encoding. (4) Attention underexploration limitation → We apply spatial attention mechanisms. This makes the motivation and relevance of each contribution explicit.

Related Work Section

1. Critical Perspective

Comment: The section is largely descriptive, resembling a tutorial rather than a critical literature review.

Response: Thank you for the helpful comment. We have substantially revised the Related Work section to adopt a more critical and analytical perspective. For each major approach (Traditional Methods, Deep Learning Methods, Siamese Networks, Attention Mechanisms), we now critically assess: (a) What problems does it solve well? (b) What are its fundamental limitations? (c) Under what conditions does it fail? We also analyze how the field has evolved and explicitly discuss why existing methods are insufficient for Chinese character-level comparison. This transforms the section from a tutorial into a critical literature review.

2. Comparative Analysis

Comment: Prior works are not compared or contrasted in terms of strengths and limitations.

Response: We appreciate the reviewer's insightful comment. We have revised the literature review to include explicit comparative analysis. For Traditional Methods: Strengths include computational efficiency and interpretability; Limitations include extensive preprocessing requirements, sensitivity to noise, and poor scalability. For Deep Learning Methods: Strengths include automatic feature learning and robustness; Limitations include data-hunger and feature entanglement issues. For Siamese Networks: Strengths include natural fit for comparison tasks; Limitations include focus on whole signatures rather than character-level. This comparative analysis clearly positions our method relative to prior work.

3. Insufficiency Explanation

Comment: The discussion does not clearly explain why existing methods are insufficient for the proposed task.

Response: Thank you for the helpful comment. We have clarified why existing methods are insufficient for Chinese character-level handwriting comparison. Traditional methods like KNN and SVM are sensitive to character rotation, variable spacing, and background noise—factors common in real-world Chinese handwriting. Standard CNNs process entire signatures, learning both handwriting style AND character structure simultaneously, leading to confusion when comparing characters with similar structures but different handwriting styles. Current Siamese networks are designed for Latin script signatures and lack mechanisms to explicitly handle character identity, making them not directly applicable to Chinese character-level comparison.

4. Connection to Proposed Approach

Comment: The connection between reviewed studies and the proposed approach is weakly articulated.

Response: Thank you for the helpful comment. We have strengthened the connection between reviewed studies and our proposed approach. Building on the observation that traditional methods suffer from limited feature learning, we adopt CNN-based feature extraction with Siamese architecture. While Siamese networks have been successfully applied to signature verification, we extend this approach to the character level, motivated by the need to reduce noise sensitivity. Inspired by feature disentanglement approaches, we explicitly provide character identity through one-hot encoding to address the limitation of standard CNNs that learn both style and structure simultaneously.

5. Missing References

Comment: Some key references relevant to attention-based similarity learning and writer verification are missing.

Response: Thank you for the helpful comment. We have added several key references including: (1) Attention-Based Similarity Learning: Shaikh et al. (2020),Hossain et al. (2025) (2) Writer Verification: Suresh et al. (2026), Raj et al. (2025); (3) Chinese Handwriting Analysis: Deng et al. (2025), Ji et al. (2022), Wang et al. (2023). These references provide a more complete and balanced background.

Attention Siamese Networks Section

1. Methodological Motivation

Comment: Although technically detailed, the section lacks a high-level methodological motivation explaining why specific architectural choices were made.

Response: We have added high-level methodological motivation for key architectural choices: (1) Siamese Architecture: Chosen because handwriting comparison is fundamentally a similarity learning problem; shared weights ensure comparable embeddings. (2) Character-Level Processing: Motivated by the observation that whole-signature methods suffer from noise in spacing, rotation, and alignment. (3) Spatial Attention: Chosen over channel attention because character images are grayscale (single channel), making channel attention less informative. (4) One-Hot Encoding: Inspired by feature disentanglement research to filter out character structure information and focus on handwriting style features.

2. Implementation Details

Comment: The section is overly implementation-focused, with excessive low-level details that could be moved to an appendix.

Response: Thank you for this valuable suggestion. We have restructured the experimental sections to improve conciseness and logical flow. Specifically, we have merged the original Section 4.3 (Implementation Details) into Section 4.2, creating a new unified section titled "Evaluation Metrics and Experimental Setup". This integration offers several advantages:

Enhanced Logical Coherence: The progression from evaluation criteria (metrics definition) to experimental validation (training setup) creates a seamless narrative flow for understanding our experimental framework.

Selective Detail Preservation: We have removed trivial hyperparameter specifications (e.g., exact learning rate, batch sizes, image dimensions) that represent standard deep learning practices, while retaining task-specific strategies critical for reproducibility, such as the 1.5:1 negative-positive sampling ratio and the contrastive loss margin (r=2.0 ).

Eliminated Redundancy: By consolidating these complementary aspects of the experimental design into one section, we avoid the fragmentation of information while maintaining sufficient detail for readers to understand and reproduce our key results.

Experiments Section

1. Additional Metrics (ROC/EER)

Comment: No additional critical metrics (e.g., ROC/EER) are discussed, which could further strengthen evaluation.

Response: Thank you for the helpful suggestion regarding additional evaluation metrics. We acknowledge that ROC curves and EER (Equal Error Rate) are valuable metrics in biometric verification systems. However, we would like to clarify the following points regarding our evaluation methodology:

(1) Standard Metrics in Handwriting Comparison Domain

In the handwriting comparison and signature verification domain, FAR (False Acceptance Rate), FRR (False Rejection Rate), and ACC (Accuracy) are the standard evaluation metrics widely adopted in the literature. These three metrics comprehensively reflect the model's performance on both positive and negative samples. Specifically:

- FAR measures the likelihood that the model incorrectly accepts an impostor as normal data, corresponding to False Positive Rate (FPR) in ROC analysis.

- FRR measures the likelihood that the model incorrectly rejects normal data, where (1-FRR) corresponds to True Positive Rate (TPR) in ROC analysis.

- ACC provides an overall measure of correct classifications.

(2) Relationship Between FAR/FRR and ROC/EER

The trade-off between FAR and FRR inherently captures the same information as ROC curves. By reporting FAR and FRR values at the operating threshold (margin r = 2.0 in our contrastive loss), we provide sufficient information to understand the model's discrimination capability. The EER (Equal Error Rate) can be estimated from our reported results:

- For CLASN: FAR = 12.72%, FRR = 10.17%, approximate EER ≈ 11.4%

- For CLASNF: FAR = 11.93%, FRR = 9.36%, approximate EER ≈ 10.6%

These EER values are competitive with recent studies. For example, Kulkarni & Wagh (2026) reported EER of 10.08% on signature verification using Siamese+MobileNetV2, while our CLASNF achieves comparable performance (~10.6% EER) on the more challenging character-level comparison task.

(3) Practical Considerations

In real-world deployment scenari

Attachment Submitted filename: ResponsetoReviewer.docx

# Peer review: Decision Letter 1

**DOI:** 10.1371/journal.pone.0353503.r003

**Authors:** Dhamyaa Abbas Nasrawi

24 Jun 2026

Character Level Attention Siamese Networks for Chinese Handwriting Comparison

PONE-D-25-66182R1

Dear Dr. Zhu,

We’re pleased to inform you that your manuscript has been judged scientifically suitable for publication and will be formally accepted for publication once it meets all outstanding technical requirements.

Within one week, you’ll receive an e-mail detailing the required amendments. When these have been addressed, you’ll receive a formal acceptance letter and your manuscript will be scheduled for publication.

An invoice will be generated when your article is formally accepted. Please note, if your institution has a publishing partnership with PLOS and your article meets the relevant criteria, all or part of your publication costs will be covered. Please make sure your user information is up-to-date by logging into Editorial Manager at Editorial Manager® and clicking the ‘Update My Information' link at the top of the page. For questions related to billing, please contact billing support.

If your institution or institutions have a press office, please notify them about your upcoming paper to help maximize its impact. If they’ll be preparing press materials, please inform our press team as soon as possible -- no later than 48 hours after receiving the formal acceptance. Your manuscript will remain under strict press embargo until 2 pm Eastern Time on the date of publication. For more information, please contact onepress@plos.org.

Kind regards,

Dhamyaa Abbas Nasrawi, Ph.D.

Academic Editor

PLOS One

Additional Editor Comments (optional):

Reviewers' comments:

Reviewer's Responses to Questions

**Comments to the Author**

Reviewer #1: All comments have been addressed

**********

2. Is the manuscript technically sound, and do the data support the conclusions?

Reviewer #1: Yes

**********

3. Has the statistical analysis been performed appropriately and rigorously?

Reviewer #1: Yes

**********

4. Have the authors made all data underlying the findings in their manuscript fully available?

The PLOS Data policy

Reviewer #1: Yes

**********

5. Is the manuscript presented in an intelligible fashion and written in standard English?

Reviewer #1: Yes

**********

Reviewer #1: (No Response)

**********

what does this mean?). If published, this will include your full peer review and any attached files.

If you choose “no”, your identity will remain anonymous but your review may still be made public.

**Do you want your identity to be public for this peer review?**  For information about this choice, including consent withdrawal, please see our Privacy Policy

Reviewer #1: **Yes:**  Mais Saad Safoq

**********

# Editor report: Acceptance letter

**DOI:** 10.1371/journal.pone.0353503.r004

**Authors:** Dhamyaa Abbas Nasrawi

PONE-D-25-66182R1

PLOS One

Dear Dr. Zhu,

I'm pleased to inform you that your manuscript has been deemed suitable for publication in PLOS One. Congratulations! Your manuscript is now being handed over to our production team.

At this stage, our production department will prepare your paper for publication. This includes ensuring the following:

* All references, tables, and figures are properly cited

* All relevant supporting information is included in the manuscript submission,

* There are no issues that prevent the paper from being properly typeset

You will receive further instructions from the production team, including instructions on how to review your proof when it is ready. Please keep in mind that we are working through a large volume of accepted articles, so please give us a few days to review your paper and let you know the next and final steps.

Lastly, if your institution or institutions have a press office, please let them know about your upcoming paper now to help maximize its impact. If they'll be preparing press materials, please inform our press team within the next 48 hours. Your manuscript will remain under strict press embargo until 2 pm Eastern Time on the date of publication. For more information, please contact onepress@plos.org.

You will receive an invoice from PLOS for your publication fee after your manuscript has reached the completed accept phase. If you receive an email requesting payment before acceptance or for any other service, this may be a phishing scheme. Learn how to identify phishing emails and protect your accounts at https://explore.plos.org/phishing.

If we can help with anything else, please email us at customercare@plos.org.

Thank you for submitting your work to PLOS One and supporting open access.

Kind regards,

PLOS One Editorial Office Staff

on behalf of

Assist.Prof.Dr. Dhamyaa Abbas Nasrawi

Academic Editor

PLOS One
